# Stem cell-derived brain-like endothelial cells to interrogate *Streptococcus pneumoniae* interaction with brain endothelium

**DOI:** 10.1080/21505594.2025.2564281

**Published:** 2025-10-03

**Authors:** Henry D. Mauser, Taryn E. Keyzer, Jessica M. Surma, Natalie G. Alexander, William D. Cutts, Sarah F. Hathcock, Kimberly H. Lackey, Daryl W. Lam, Justin A. Thornton, Nadine Vollmuth, Brandon J. Kim

**Affiliations:** a Department of Biological Sciences, University of Alabama, Tuscaloosa AL, USA; b Department of Biological Sciences, Mississippi State University, Starkville MS, USA; c Department of Biological Sciences, University of Texas at Dallas, Richardson, TX, USA; d Department of Microbiology, Heersink School of Medicine, University of Alabama at Birmingham, Birmingham AL, USA; e Center for Convergent Biosciences and Medicine, University of Alabama, Tuscaloosa AL, USA; f Alabama Life Research Institute, University of Alabama, Tuscaloosa AL, USA

**Keywords:** *Streptococcus pneumoniae*, blood-brain barrier, pneumococcal meningitis, brain endothelial cells, iPSC-BEC

## Abstract

*Streptococcus pneumoniae* (pneumococcus) is an opportunistic pathogen that remains the leading cause of bacterial meningitis worldwide. For meningitis to occur, pneumococcus must breach the blood-brain barrier (BBB), a highly specialized network of brain endothelial cells that comprise the microvasculature of the brain. Here, we report the use of human induced pluripotent stem cell-derived brain-like endothelial cells (iBECs) to model the BBB during pneumococcal infection. iBECs were infected with the *S. pneumoniae* strain TIGR4. Adherence assays showed that pneumococcal adherence to iBECs was a saturable process. Moreover, deletion of two pneumococcal adhesins resulted in an adherence defect, supporting a receptor-mediated interaction between pneumococcus and iBECs. Next, the integrity of several tight junction components was assessed via western blot and RT-qPCR, revealing the loss of abundance and expression in iBECs during infection with pneumococcus. Simultaneously, the expression of *VEGFA* and the tight junction repressor *SNAI1* was upregulated. Semi-automated analysis of junction images also demonstrated a loss of ZO-1 and occludin continuity during pneumococcal infection. Consistent with these findings, the loss of TEER and the increase in barrier permeability were observed in pneumococcus-infected iBECs. The toxin pneumolysin (Ply) was important for this disruption, as the loss of Ply in pneumococcus partially arrested the reduction of TEER and the increase in permeability. Finally, RT-qPCR showed that pneumococcus was sufficient to upregulate a panel of inflammatory cytokines in iBECs. Taken together, these findings show that pneumococcus interacts with and disrupts iBECs during infection, supporting iBECs as an important model for studying pneumococcus-BBB interactions.

## Introduction

*Streptococcus pneumoniae* (pneumococcus or *Spn*) is a gram-positive opportunistic pathogen that frequently colonizes the mucosa of the upper respiratory tract in young children and adults. In certain individuals, *S. pneumoniae* may gain access to the sterile regions of the lower respiratory tract and cause pneumonia; alternatively, if pneumococcus crosses the nasal or alveolar epithelium and enters the bloodstream, it may cause invasive pneumococcal disease manifesting as sepsis and/or meningitis [1]. Despite the efficacy of the pneumococcal conjugate vaccines (PCV) and pneumococcal polysaccharide vaccines (PPSV), *S. pneumoniae* remains the leading cause of bacterial meningitis/meningoencephalitis worldwide accounting for over 60% of deaths in all cases of bacterial meningitis [2–4]. *S. pneumoniae* particularly impacts children under the age of 5 and adults over the age of 65 [5,6]. Without treatment, pneumococcal meningitis is routinely fatal, and even among groups who receive intensive care, mortality is 10–20% [2,7]. Additionally, many patients surviving meningitis suffer long-term neurological sequelae including hearing loss and cognitive impairment [7–9].

Bacterial meningitis/meningoencephalitis refers to the severe inflammation of the brain and central nervous system (CNS) due to infection. Indeed, pneumococcal invasion of the CNS is accompanied by a severe inflammatory response characterized by the mass infiltration of neutrophils into the meninges and parenchyma of the brain [10,11]. These activated neutrophils, other innate immune components such as macrophages and brain microglia, and pneumococcal products such as H_2_O_2_ and cell wall components produce overwhelming inflammation in the brain [12–14]. The resulting neuronal death, edema, and increase in intracranial pressure prove lethal if left unresolved.

Prior work has demonstrated several viable routes for pneumococcus to enter the brain. Direct entry into the brain from the nasopharynx by crossing the cribriform plate may occur, particularly during surgery or traumatic injury, but typically pneumococcus enters the brain from the bloodstream [15,16]. If from the bloodstream, pneumococcus must either cross the blood-cerebrospinal fluid (CSF) barrier at the choroid plexus or the specialized endothelium of the blood-brain barrier (BBB) and meningeal blood-CSF barrier (mBCSFB) [17,18]. The BBB and mBCSFB comprise the microvasculature of the CNS and primarily consist of a single layer of highly specialized brain endothelial cells (BECs) which physically restrict the entry of circulating pathogens, leukocytes, toxins, and other large molecules into the CNS. These cells are characterized by several hallmarks which distinguish them from traditional vascular endothelium, including the presence of complex tight junctions, low rates of endocytosis, expression of various efflux transporters and nutrient importers, and in the case of the BBB, suppression of surface-expressed leukocyte adhesion molecules and chemoattractants [19–22]. These characteristics of the BBB help support the immune privilege of the brain, protect the CNS from inflammation, and maintain homeostasis. The BECs of the BBB are additionally supported by associated pericytes and astrocyte foot processes that help regulate the function of BECs and confer additional barrier integrity [23]. The entry of pathogens into the brain and subsequent inflammation during bacterial meningitis may represent a loss of function at the BBB.

To investigate the interaction between pneumococcus and the BBB, prior research has utilized several *in vivo* and *in vitro* systems to model the BBB. Rodent and rabbit models have been utilized most frequently to study pneumococcal meningitis *in vivo* [24]. Most rat and rabbit models rely on an intracisternal route to induce meningitis, and are thus most useful for studying the effects of rather than the events resulting in meningitis [24–29]. Several intranasal and intraperitoneal routes of infection have been established in infant and adult rodent models which mimic the natural route of infection and the progression of pneumococcal colonization to meningitis [24,30–36]. Unlike intracisternal models in which the brain is directly inoculated, these models may be used to study how *S. pneumoniae* reaches the CNS, including how the bacterium interacts with and traverses the blood-brain barrier prior to causing meningitis. Intravenous routes of infection have also been utilized to induce meningitis in mice [16,32,37–39]. Although these infection models incompletely represent the progression of pneumococcal disease, they likewise enable the study of pneumococcal entry into the CNS. Frequently utilized *in vitro* models are primary immortalized human or murine brain endothelial cells or primary human BECs [40–42]. Due to the limited availability of primary human BECs, primary immortalized cell lines are more commonly used for human *in vitro* model systems. Human brain microvasculature endothelial cells (hBMECs) and human cerebral microvascular endothelial cells (hCMEC/D3s) comprise the human primary immortalized cell lines previously used to study pneumococcal interaction with BECs [43–45].

Recently, brain-like endothelial cells have been generated from human induced pluripotent stem cells and have been utilized to model BECs [21]. These induced pluripotent stem cell-derived brain-like endothelial cells (iPSC-BECs or iBECs) reproduce key hallmarks of the blood-brain barrier such as high tight junction expression, including elevated claudin-5 expression, as well as high BEC transporter GLUT-1 and P-glycoprotein expression [46]. Furthermore, iBECs exhibit remarkably high TEER values ranging around 3000 Ωxcm^2^, which approaches 100 times higher than typical values obtained for hBMECs and hCMEC/D3s [46–48]. The iBEC model has been utilized to study a variety of meningeal pathogens such as *Neisseria meningitidis*, *Streptococcus agalactiae*, Coxsackievirus B3, Zika virus, and COVID-19 [20,49–57]. Previously, no study has investigated the interaction between *S. pneumoniae* and the BBB through the lens of an iBEC model.

In this study, we generated iBECs and infected them with the neurotropic *S. pneumoniae* strain TIGR4, obtained from Dr. Justin Thornton [58,59]. We observed that *S. pneumoniae* associated with iBECs in a specific manner, and that adherence to iBECs was dependent on CbpA and RrgA expression, two previously characterized adhesins. Together, these data suggested receptor-mediated interaction between pneumococcus and iBECs, in support of previous work. Moreover, we found that *S. pneumoniae* induced the dysregulation of iBEC tight junction components ZO-1, occludin, and claudin-5 during infection. Not only was the expression and abundance of these tight junction proteins decreased during infection, but semi-automated digital analysis of junction images suggested that pneumococcal infection reduces junction continuity. Consistent with these findings, the tight junction repressor gene *SNAI1* was found to be upregulated during infection. Additionally, TEER experiments showed that pneumococcus damages the barrier function of iBECs, which may partially depend on secretion of the pneumolysin toxin. Upon assessing a panel of inflammatory cytokines, we found that expression of inflammatory markers was ubiquitously increased during infection. Taken together, these findings strongly support iBECs as a valuable model for studying *S. pneumoniae* interaction with BECs.

## Materials and methods

### Bacterial strains and culture

*S*. *pneumoniae* strains used were TIGR4 [59,60] or mutants from an identical TIGR4 background. Strains were either cultured in THY broth (Todd Hewitt Broth + .5% yeast extract) or on 5% sheep’s blood agar plates (Todd Hewitt Broth + 1.5% agar + 5% sheep’s blood) and grown at 37 °C + 5% CO_2_. Frozen stocks were created by growing the bacteria to an OD_600_ of .4 and freezing at 80 °C in 24% glycerol. Additionally, to create starter cultures for use in experiments, strains were grown to an OD_600_ of .4 and frozen at −80 °C in 15% glycerol in 1 mL aliquots. Prior to an experiment, a 1 mL aliquot was thawed and diluted in 4 mL of fresh THY and allowed to grow to an OD_600_ of .4–.6 before use. The relationship between OD_600_ and CFU/mL of each strain was determined prior to use in experiments (data not shown).

### Generation of pneumococcal mutants

In the case of Δ*cbpA* and Δpilus mutants, 500–1500 base pairs of flanking DNA were amplified from the upstream and downstream regions of the respective operons. An erythromycin resistance cassette was amplified from the pHY304 plasmid. PCR was performed using Q5® High-Fidelity DNA Polymerase (NEB cat#M0515) or Phusion Green Hot Start II High-Fidelity PCR Master Mix (Thermo Scientific cat#F566L). All primers used are listed in Supplement Table S2. All PCR products used for assembly or transformation were verified by size by running at 180 volts on a 1% agarose TAE gel alongside a 1 kb DNA ladder (NEB cat#N3232S); these products were purified using the Wizard® SV Gel and PCR Clean-Up System (Promega cat#A9281). The upstream, downstream, and erythromycin cassette products were assembled using the NEBuilder® HiFi DNA Assembly Master Mix (NEB cat#E2621S) according to the instructions provided. Similarly, for construction of the JS1 (capsule negative TIGR4), approximately 750–800 base pairs of DNA flanking each side of type 4 capsule locus within the *dexB* and *aliA* genes was amplified by PCR using Q5 polymerase and fused with an erythromycin (ErmB) cassette using splicing by overlap extension (SOEing) PCR and subsequent 3kB products were verified by DNA electrophoresis and used for transformations. Individual PCR products and final SOEing products were purified using the Zymo DNA Clean and Concentrator-25 kit (Zymo cat#D4006). To transform pneumococcus, TIGR4 was grown in sterile filtered competence media (THY + .2% glucose + .2% BSA) to an OD_600_ of .6, and then back-diluted 1:50 in prewarmed comp media + 1:100 CaCl_2_. Competence stimulating peptide 2 (CSP-2) (Anaspec cat#AS-63877) was added at 1:100 to induce competence, and the mixture was incubated for 12 minutes at 37 °C. Transforming DNA was then added at 5–10 μg/mL, and the mixture was incubated for 2 hours at 37 °C. 100 μL was spread onto selection plates (5% sheep’s blood agar +.5 μg/mL erythromycin) and incubated overnight at 37 °C + 5% CO_2_. Colonies were verified by colony PCR, grown in THY +5 μg/mL erythromycin, and kept as frozen stocks at −80 °C in 24% glycerol. Additionally, mutant stocks were PCR verified after freezing down. Δ*ply* and Δ*spxB* mutants were provided by Dr. Justin Thornton (Mississippi State University) and were created by a similar method as above as previously described [61,62]. To ensure that all mutants were in an identical TIGR4 background and passage number, the flanking DNA + erythromycin resistance cassettes of these mutants were amplified together using outside primers. The resulting product was verified, purified, and used for transformation as previously described to generate the Δ*ply* and Δ*spxB* mutants. All primers used are listed in Supplement Table S2.

### Generation and validation of iPSC-derived brain-like endothelial cells

iBECs were differentiated from the iPS(IMR90)-4 cell line (Wicell hPSCReg ID WISCi004-B) as previously described [21,46,50,51,63]. Briefly, IMR90s were maintained in Stemflex^TM^ Basal Medium (Thermo cat#A3349401) and differentiated in Unconditioned Media (DMEM/F12 (Thermo cat#11320033), KOSR (Thermo cat#10828028), NEAA (Thermo cat#11140050), GlutaMAX (Thermo cat#35050061, β-mercaptoethanol) for 6 days. After selectively expanding the endothelial cell population for two additional days in EC media (hESFM (Thermo cat#11111044), B27 (Thermo cat#17504044), with or without bFGF (PeproTech cat#100-18B-50UG) and retinoic acid), iBECs were purified by seeding onto Collagen IV and fibronectin coated plates. Two days after purification, the iBECs reach maximum TEER values and are ready for experiments. To validate the differentiation of iBECs, various markers were immunolabelled as previously described [51] and imaged via immunofluorescent microscopy. Briefly, cells in a 48-well plate were washed 3x with PBS and fixed for 15 minutes in 100% ice-cold methanol. Cells were then washed 3x with PBS and blocked for 1 hour in blocking buffer (10% FBS in PBS). After blocking, primary antibody diluted in blocking buffer was applied overnight at 4 °C. All antibodies and dilutions used are listed in Supplement Table S1. The next day, cells were washed 3x with PBS and incubated with a fluorescent secondary antibody (anti-mouse Alexa fluor 488, Thermo cat#A11001) for 1 hour at RT. Cells were then washed, incubated with DAPI for 15 minutes, and imaged. All images were captured on an Eclipse Ti2-E inverted microscope (Nikon).

### Infection assays

After growing bacterial strains as previously reported, bacteria were spun down at 3220xg for 5 minutes and resuspended in 200 μL of PBS. Each strain was then normalized in PBS to its respective OD_600_ to achieve 1x10^8^ CFU/mL. The inoculum or an equivalent amount of PBS was then diluted into prewarmed EC-/- media to achieve the desired MOI or mock condition. The iBEC plates or transwells were then aspirated of media and infected with the prepared EC-/-. We estimate 1x10^5^ cells per well for a 24-well plate, and 8x10^4^ cells per 24-well transwell. Thus, to achieve an MOI of 10 on a 24-well plate, for example, bacteria at 1x10^8^ were diluted 1:25 in EC-/-, and 250 μL of the inoculated EC-/- were then used to infect each well. Calculations for different sized plates were scaled appropriately to achieve the same target MOIs. For TIGR4 WT vs mutant adherence assays, cells were pretreated for 2 hours with TNF-α (10 ng/mL) (Biolegend cat#570104) prior to infection. After infection, plates were incubated at 37 °C + 5% CO_2_ for 30 minutes, then aspirated and washed 3x with PBS. 100 μL of 0.25% trypsin-EDTA were then added to each well and the plate was incubated at 37 °C + 5% CO_2_ for 10 minutes. 400 μL of 0.025% triton-x in PBS were added to each well and pipetted up and down vigorously 30x to lyse cells. The contents of each well were then serially diluted in PBS and drip-plated on either THY or 5% sheep’s blood agar plates. Plates were incubated overnight at 37 °C + 5% CO_2_ and counted the following day.

### Pneumococcal growth curves

Pneumococcal strains were grown from starter cultures and normalized in PBS to achieve 1x10^8^ CFU/mL as previously reported. Bacteria at 1x10^8^ were then diluted 1:25 in EC-/- and 250 μL were transferred to each well of a lidded 96-well plate. Bacteria were grown in the 96-well plate for 16 hours at 37 °C in a SpectraMax iD3 Microplate Reader (Molecular Devices), which measured absorbance at 600 nm every .5 hours.

### Protein collection and western blot

Cells were infected as reported above and incubated at 37 °C + 5% CO_2_ for 3.5 hours. At the time-point, the media was aspirated and 100 μL of RIPA buffer + 1:100 Halt™ Protease Inhibitor Cocktail (100x) (Thermo cat#78438) were added. A 1000 μL micropipette tip was used to scratch the bottom of each well and detach the cells. The lysates were collected and homogenized using a syringe and 29 gauge 12.7 mm needles, then spun down at 13000xg for 15 minutes at 4 °C to pellet cell debris. The supernatant was collected and used for future assays. To determine the protein concentration of each sample, the Pierce™ BCA Protein Assay Kit (Thermo cat#23255) was utilized. For the western blot, using identical micrograms of protein, up to 30 μL of each sample were combined with 10 μL of NuPAGE™ LDS Sample Buffer (4X) (Invitrogen cat#NP0008) and run at 140 V for 40 minutes on NuPAGE™ Bis-Tris Mini Protein Gels (Invitrogen cat#NP0323BOX) along with SeeBlue™ Plus2 Pre-stained Protein Standard (Invitrogen cat#LC5925). The gels were then transferred at 300 mAmps for 90 minutes to nitrocellulose Amersham™ Protran® Premium Western blotting membranes (Millipore Sigma cat#GE10600096). Gel electrophoresis and membrane transfers were performed using the Mini Gel Tank and Blot Module Set (Thermo cat#NW2000). After transferring, Ponceau S stain was applied to the membrane and imaged to visualize total protein content. The membrane was then washed briefly in TBST and blocked in 5% milk + TBST for at least 1 hour. Primary antibodies at appropriate dilutions in 5% milk + TBST were applied and the membrane was allowed to shake at 4 °C overnight. The following day, the membrane was washed 3x for 5 minutes with TBST, and then secondary antibodies in 5% milk + TBST were applied and the membrane was allowed to shake at RT for 1 hour. After washing 3x for 5 minutes with TBST again, the membrane was imaged on an iBright™ 1500 Imaging System (Thermo Fisher). Bands were visualized using SuperSignal™ West Pico PLUS Chemiluminescent Substrates (Thermo cat#34580). All primary and secondary antibodies and the dilutions used are listed in Supplement Table S1.

### Cytotoxicity assays

The cytotoxicity of pneumococcus toward iBECs was measured using the LDH-Cytox™ Assay Kit (Biolegend cat#426401). iBECs in a standard 96-well plate were infected as previously described with 62.5 μL of EC -/- containing 2.5x10^5^ CFU TIGR4 or JS1 to achieve an MOI of 10. 10 μL of lysis buffer was added to the lysis control wells 30 minutes prior to the end of the infection time. At the end of the infection time, 100 μL of working solution was added to each well and incubated at 37 °C + 5% CO_2_ for 30 minutes. 50 μL of stop solution were added to each well, and the absorbance of each well was measured at 490 nm using a SpectraMax iD3 Microplate Reader (Molecular Devices). The absorbance values for each sample were subtracted from a background control value, and then the % cytotoxicity of each well was calculated via the following equation: $\frac{\mathrm{value}-\mathrm{mock}\;\mathrm{avg}}{\mathrm{lysis}\;\mathrm{control}\;\mathrm{avg}-\mathrm{mock}\;\mathrm{avg}}\times 100$.

### RNA isolation and RT-qPCR

Cells were infected as reported above and incubated at 37 °C + 5% CO_2_ for 3.5 hours. At the time-point RNA was isolated and then purified using materials and instructions provided in the Machery-Nagel NucleoSpin RNA Kit (Fisher cat#740955.250). Purified RNA concentrations were measured using a Nanodrop 2000 spectrophotometer (Thermo Scientific), and then identical amounts of RNA were converted into cDNA using a qScript cDNA synthesis kit (Quantabio cat#95047–500). The resulting cDNA was diluted 1:10 in nuclease-free water prior to use for qPCR. Gene expression was quantified via qPCR using PowerTrack SYBR Green Master Mix (Thermo cat#A46109) and was measured by a QuantStudio3 (Thermo Fisher). ΔΔCT calculations were performed using GAPDH as a housekeeping gene. Data are shown as fold change over mock infected cells. All primers used for qPCR are listed in Supplement Table S2.

### Immunostaining and fluorescent microscopy

For comparative tight junction immunostaining, iBECs were infected with mock or TIGR4 for 3.5 hours as described above. After infection, iBECs were stained using the procedure described above. 5 images were captured per well, and images were captured in the same 5 regions of each well. For imaging of *Spn* interacting with pIgR and CD31, cells were grown on 12 mm glass coverslips in a 24-well plate. Cells were infected with mock or TIGR4 at an MOI of 10 for 30 minutes as described above. After infection, iBECs were washed 3x with PBS, then fixed, blocked, and stained using the procedure described above. A combination of anti-*Streptococcus pneumoniae* and anti-pIgR or anti-CD31 antibodies was used. After the staining procedure was complete, the glass coverslips were inverted, and fixed in place on glass slides. Cells and cell-associated *S. pneumoniae* were then imaged using immersion oil microscopy. Representative images of *S. pneumoniae* interacting with iBECs were captured. All antibodies and dilutions used are listed in Supplement Table S1.

### TEER and permeability assays

iBECs were seeded on 6.5 mm 24-well transwells (Corning Cat#3470) as described above. For TEER assays, TEER was measured just before infection and reported as hour 0, and then transwells were infected as described above. TEER values were measured at designated time points. For permeability assays, iBECs were seeded on transwells and infected as just described. At 5 or 7 hours, the media in apical transwell compartment was replaced with 10 μM sodium fluorescein (NaF) (Thermo Cat#J61549.22), and NaF permeability was assessed as previously described [63].

### Junction image analysis

To assess the continuity of tight junctions visualized by immunofluorescent imaging, the Junction Analyzer Program (JAnaP) was used, available for download at https://github.com/StrokaLab [64]. JanaP enables the user to manually trace the imaged tight junction around each cell, and then generate values for a variety of parameters such as size, shape, or percent continuity. Three images per well were randomly selected for analysis and every non-adjacent cell within an image was manually waypointed in JAnaP. After waypointing, a representative cell from the mock condition was selected for thresholding in Python Jupiter Notebook, and an appropriate filter intensity cutoff was selected to display junctions but eliminate background noise in each image. JAnaP was then used to analyze all selected cells and quantify cell morphology parameters along with junction characteristics such as % coverage and % continuity, or whether a non-continuous junction was punctate or perpendicular. % continuity measurements were graphed, but all JAnaP measurements are available in the supplementary materials.

### Statistics

ROUT (Q = 1%) outlier test was applied to data sets and outliers were removed where identified. All data were analyzed using one-way and two-way ANOVA analyses for data sets with three or more conditions, or an unpaired student’s *t*-test for data sets with two conditions. Data with a non-normal distribution were analyzed via a Mann-Whitney-Wilcoxen test. Significance is defined as *p* < .05. All experiments were conducted in technical and biological replicate unless otherwise specified. Significance was determined by analyzing the combination of all technical replicates. Graphpad Prism 10.30.0 was utilized to generate all graphs and statistics.

## Results

### Pneumococcus associates with iBECs

**Figure 1.**
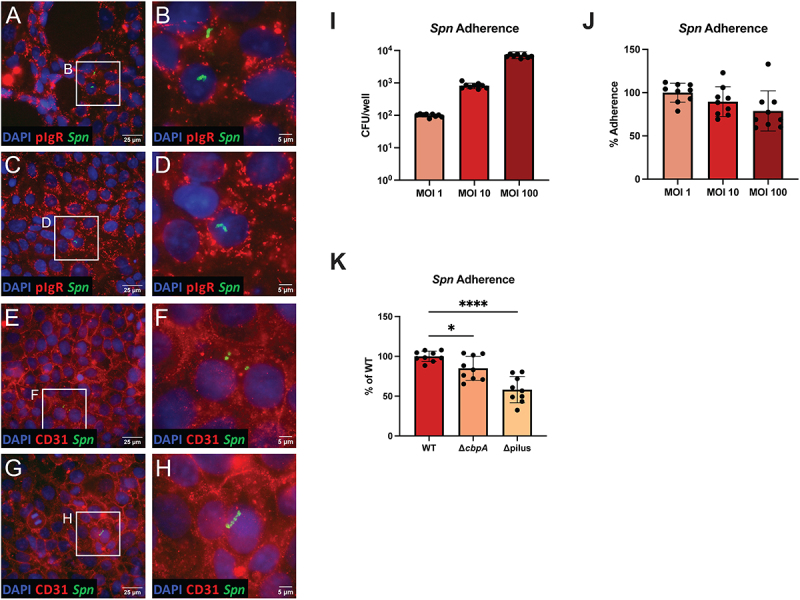
Pneumococcal interaction with and adherence to iBECs. (A-H) Representative composite immunofluorescent images of iBECs infected with TIGR4 (*Spn)* for 30 min at an MOI of 10 with full-scale (left) and cropped (right) images. Scale bars and legends are shown. Representative images were captured from a single iBEC differentiation to visualize the nuclei via DAPI (blue), *Spn* (green), and pIgR or CD31 (red). (I-K) Adherence of JS1 (*Spn*) (I-J) or WT TIGR4 and pilus- or CbpA-deficient mutants (K) quantified at 30 min P.I. at an MOI of 10. Data for the adherence assays are normalized to their respective mock control and are displayed as the mean value of all replicates, comprised of technical triplicates from three independent iBEC differentiations. *n* = 9. Error bars represent SD. One-way ANOVA was utilized to determine significance between WT TIGR4 and the Δ*cbpA* and Δpilus mutants. *p < .05, **p < .01.

Previous work has utilized immortalized human brain endothelial cells to show that pneumococcus interacts with and disrupts the blood-brain barrier; however, many of these immortalized cell lines lack defining BEC properties [18,39,48,65]. Since iPSC-BECs have never been used to study pneumococcus, we sought to determine whether iBECs could be utilized to model pneumococcus-BBB interactions. iBECs were differentiated as established by previous work [46,50,51,63] and validated by demonstrating the presence of several endothelial-specific markers and key hallmarks of BECs such as CD31 (PECAM-1), VE-cadherin, P-glycoprotein, and claudin-5 (Fig. S1A-G). Since adherence to brain endothelial cells is typically a prerequisite for bacterial disruption or invasion of the blood-brain barrier, we first sought to determine whether pneumococcus adheres to iBECs. Moreover, during human pneumococcal meningitis, pneumococcus has been found to adhere to BECs by binding the surface expressed proteins CD31 and pIgR [66]. Thus, to characterize the interaction between pneumococcus and iBECs, we first infected confluent iBEC monolayers with the wildtype *S. pneumoniae* strain TIGR4 at an MOI of 10 CFU/cell for 30 minutes to permit adherence. Then, after washing repeatedly with PBS to remove non-adherent bacteria, we performed immunostaining procedures to visualize CD31, pIgR, and pneumococcus. We found that pneumococcus does indeed adhere to iBECs, and typically appeared as isolated diplococci or in small chains or clumps (Figure 1(A-H)). However, direct colocalization of pneumococcus with either CD31 or pIgR could not be discerned from these images. Thus, to quantitatively assess pneumococcal adherence, we infected iBEC monolayers as described above and performed adherence assays. Since previous work has shown that the pneumococcal capsule limits adherence [67,68], for this assay we used JS1, an unencapsulated derivative of TIGR4, to more clearly elucidate small changes in adherence. When we infected iBECs with increasing MOIs of JS1 we observed that the total number of adherent bacteria increased with increasing MOI (Figure 1(I)). However, when adherence was quantified as a percent of the MOI, it was observed that % adherence slightly decreased with increasing MOI (Figure 1(J)). While differences between MOI 1 and MOI 10 and MOI 10 and MOI 100 were not significantly different (*p* = 0.4441 and *p* = 0.4191, respectively), the difference between MOI 1 and MOI 100 was significant (*p* = 0.0479), indicative of a decreasing trend in % adherence (Figure 1(J)). This suggests that pneumococcal adherence to iBECs is a saturable process, supporting that the interaction is receptor-mediated, consistent with previous work in other models [39,44,65,66,69]. Pneumococcus was not cytotoxic to iBECs at an MOI of 10 or 100 at 2 hrs P.I., supporting that the observed phenotype is not due to cell death (Fig. S2A). To further investigate which receptors pneumococcus interacts with and what virulence factors pneumococcus utilizes in the process, we generated CbpA- and pilus-deficient mutants in TIGR4. Choline-binding protein A (CbpA, also referred to as PspC and SpsA) was first identified as a crucial adhesin for pneumococcal colonization of the nasopharynx and the lung [70]. Simultaneously, CbpA was identified as a novel pneumococcal surface protein that binds the secretory component (SC) of secretory immunoglobulin A (SIgA) [71]. As the SC derives from the polymeric immunoglobulin receptor (pIgR) which facilitates transport of polymeric IgA across epithelium, later work demonstrated that pneumococcus may bind to the SC component of pIgR to facilitate adherence and invasion across nasopharyngeal epithelium [72,73]. More recently, CbpA has been shown to bind pIgR in human BECs at the BBB [66]. Likewise, the pneumococcal type-1 pilus is a heterotrimeric protein identified to be crucial for adherence in the lung and nasopharynx [74,75]. The pilus subunit RrgA has been shown to bind both pIgR and CD31 in human BECs [66]. We performed adherence assays with the CbpA- and pilus-deficient mutants. Prior work shows that immune activation of BECs facilitates TIGR4 adherence and invasion, so here we activated with TNF-α prior to infection as previously described [37,43,65]. As anticipated, we found that the loss of either CbpA or the pilus significantly inhibited adherence to iBECs (Figure 1(K)), consistent with previous findings in other models [65,66,76]. To ensure that this phenotype was not the product of a growth defect, we performed a growth curve to compare wildtype with the two mutants (Fig. S3A). Although the Δpilus mutant exhibited a slight lag in growth relative to Δ*cbpA* or WT which emerged around hour 4, both mutants exhibited normal growth and reached the same OD_600_ as WT (Fig. S3A). These data may suggest that CD31 and pIgR contribute to pneumococcal interaction with iBECs, although CbpA and RrgA can also bind other host receptors such as the laminin receptor, collagen, fibronectin, and vitronectin [65,77–80].

### Pneumococcus decreases expression and abundance of tight junction components

**Figure 2.**
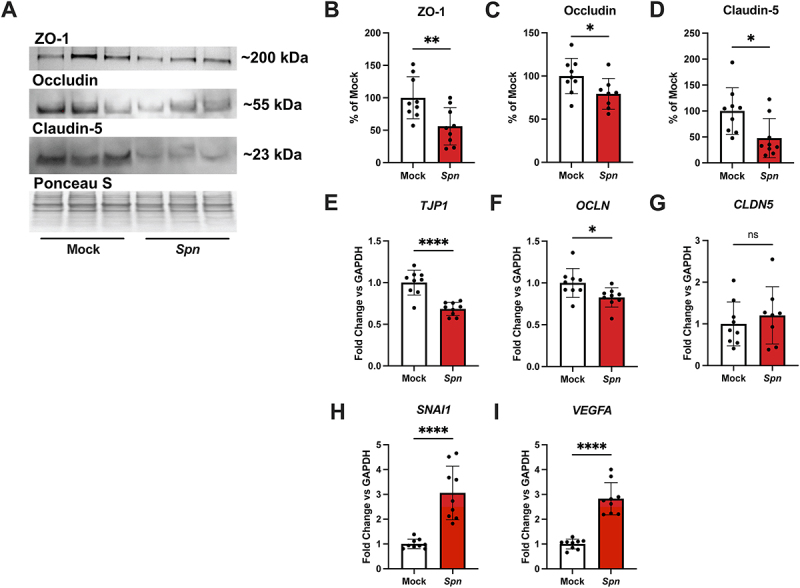
iBEC tight junction regulation during pneumococcal infection. iBECs were treated for 3.5 hours with mock or TIGR4 (*Spn)* at an MOI of 10. (A) Representative western blot featuring ZO-1, occludin, and claudin-5 with and without infection. Ponceau S was visualized as a loading control. (B-D) Quantification via densitometry of western blots from three independent iBEC differentiations. Values were corrected against Ponceau S and normalized to mock. (E-I) RT-qPCR ΔΔCT values representing changes in expression of *TJP1, OCLN, CLDN5, SNAI1*, and *VEGFA* during infection. *GAPDH* was used as the housekeeping gene. Data are displayed as the mean of all replicates, comprised of technical triplicates from three independent iBEC differentiations. *n* = 9. Error bars represent SD. ROUT (Q = 1%) outlier test was applied to data sets and one outlier was removed from (C) and (G). Outlier values for (C) *Spn* and (G) *Spn* were 203.15 and 9.75, respectively. For (C) *Spn* and (G) *Spn*, *n* = 8. Student’s t-test was utilized to determine significance between mock and *Spn*. *p < .05, **p < .01. ****p < .0001.

After demonstrating specific interaction between pneumococcus and iBECs, we investigated whether pneumococcus would disrupt the iBEC barrier via tight junction dysregulation. The selectivity of the BBB is largely due to the presence of tight junction complexes that connect adjacent cells and limit paracellular transport [81,82]. Previous work in other models suggests that pneumococcus can specifically downregulate or degrade tight junction components such as ZO-1 and occludin in both brain endothelial cells and alveolar epithelial cells, facilitating tissue invasion [43,83–85]. Additionally, claudin-5, a highly expressed tight junction at the BBB, has been shown to be downregulated in pneumococcus-infected lung capillaries [86]. Thus, we chose to evaluate the abundance and expression of ZO-1, occludin, and claudin-5 during the infection of iBECs. iBEC monolayers were infected with mock or TIGR4 as described at an MOI of 10 for 3.5 hours, and then protein or RNA was isolated and analyzed via western blot or RT-qPCR, respectively. Upon observation, western blotting suggested that ZO-1, occludin, and claudin-5 were each reduced in abundance in the infected condition (Figure 2(A)). Quantification of the western blots via densitometry revealed that that all three proteins exhibited a significant loss of protein abundance (Figure 2(B-D)). To ensure that the loss of each protein was not due to cytotoxicity of the bacterial inoculum, we assessed the percent cytotoxicity of the infection condition. Initially, we showed at 2 hours that neither an MOI of 10 or 100 was cytotoxic to iBECs (Fig. S2A). Although an MOI of 100 at 3.5 hours did result in significant cytotoxicity in iBECs, the MOI of 10 for the same amount of time resulted in no significant cytotoxicity (Fig. S2B). This indicates that while pneumococcus is cytotoxic to iBECs at higher MOIs for long timepoints, the iBECs are still viable under the conditions used to assess protein expression and abundance. To ensure that the observed loss of protein was specific to those proteins, and not a universal downregulation of protein production due to infection, we blotted for p53, a protein with a swift turnover rate. If pneumococcus were suppressing all protein production, we would expect to see the loss of a protein with a high turnover rate like p53. While a total loss of p53 was observed at an MOI of 100 at 3.5 hours, there was no significant difference in p53 abundance at an MOI of 10 at the same timepoint (Fig. S4A-B). Finally, we assessed the expression of *TJP1, OCLN*, and *CLDN5* – the genes encoding ZO-1, occludin, and claudin-5, respectively – under mock or TIGR4 infected conditions. Consistent with the previous results, expression of *TJP1* and *OCLN* were significantly downregulated under infected conditions (Figure 2(E,F)), although *CLDN5* expression did not appear to be impacted (Figure 2(G)). Since we observed downregulation of tight junctions, we also assessed the expression of *SNAI1*, a tight junction transcriptional repressor [87,88]. *SNAI1* upregulation has been observed during pneumococcal infection of both brain endothelial and lung epithelial models in conjunction with loss of tight junction components such as ZO-1 [43,84,89]. As anticipated, we observed *SNAI1* significantly upregulated during infection (Figure 2(H)), suggesting that pneumococcus potentiates *SNAI1* activation in iBECs as a prerequisite for tight junction downregulation. Furthermore, *VEGFA* upregulation was also observed during infection (Figure 2(I)). *VEGFA* is the gene encoding a vascular endothelial growth factor responsible for regulating angiogenesis in vascular endothelium. *VEGFA* upregulation in conjunction with tight junction degradation has been observed during many different disease states of the BBB including pneumococcal infection [90–93]. While VEGF-A is not a direct regulator of tight junction components as is SNAIL1, its upregulation during pneumococcal infection in iBECs correlates with the observed loss of tight junction components. These data demonstrate that iBECs lose tight junction components during pneumococcal infection.

### Pneumococcus dysregulates tight junction organization and continuity in iBECs

**Figure 3.**
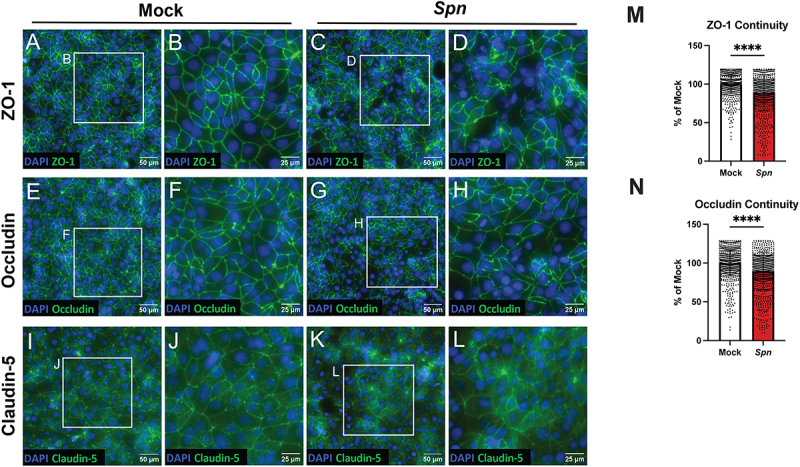
iBEC organization and continuity during pneumococcal infection. iBECs were treated for 3.5 hours with mock or TIGR4 (*Spn)* at an MOI of 10. (A-L) iBECs were stained and imaged via immunofluorescent microscopy to visualize ZO-1, occludin, or claudin-5 (green) and the nuclei via DAPI (blue). Representative composite immunofluorescent images of iBECs with full-scale (left) and cropped (right) images for both mock and *Spn*-infected conditions. Scale bars are shown. (M-N) Percent continuity of ZO-1 and occludin expressed as a percent of mock, as characterized by JanaP. Data are represented as the percent continuity of individual cells from all images. 9 images were analyzed per condition, comprised of technical triplicates from three independent iBEC differentiations. For ZO-1 mock and *Spn*, *n* = 927 and *n* = 992, respectively. For occludin mock and *Spn*, *n* = 988 and *n* = 915, respectively. Error bars represent SD. Mann-Whitney-Wilcoxen test was utilized to determine significance between mock and *Spn*. ****p < .0001.

In addition to showing loss of tight junction abundance and expression, we next investigated whether infection with pneumococcus would produce an observable loss of tight junction organization or continuity. In prior work using human lung tissue, pneumococcal infection resulted in areas of broken junctional bands [86], and we hypothesized that a similar phenotype would present in infected iBECs. Thus, we infected iBEC monolayers with TIGR4 at an MOI of 10 for 3.5 hours, then performed the described staining procedures to visualize cell nuclei and either ZO-1, occludin, or claudin-5 [51]. We observed local areas of disruption in ZO-1 and occludin where junction bands were broken or absent, whereas junctions in the mock infected monolayers remained continuous (Figure 3(A-H)). We did not observe a difference between mock and infected for claudin-5 (Figure 3(I-L)). To determine whether the observed disruption of ZO-1 and occludin in the infected condition was significant, we utilized the Junction Analyzer Program (JAnaP) to quantify tight junction continuity as previously described [64,94]. After analyzing all images via JanaP as described, the quantifications showed that the continuity of both ZO-1 and occludin was significantly downregulated in the infected condition (Figure 3(M,N)). This finding is consistent with our previous data and supports previous work conducted in other cell types. Moreover, we are the first to demonstrate pneumococcal alteration of junction phenotype in the iBEC model, which possesses more complete tight junctions than immortalized BECs *in vitro*.

### Pneumococcus disrupts the barrier function of iBECs

**Figure 4.**
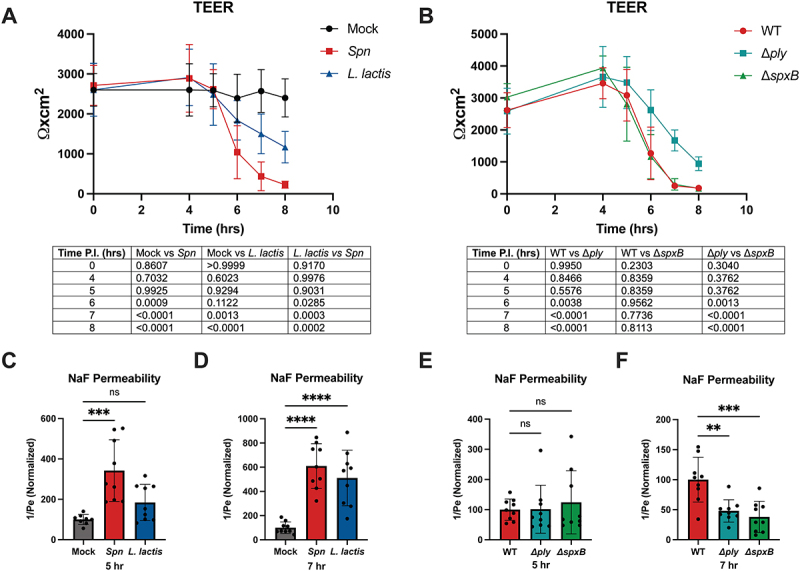
iBEC barrier function during pneumococcal infection. TEER and NaF permeability measurements of iBEC monolayers cultured on transwell inserts, and treated with mock, TIGR4, or *L. lactis* (A, C-D), or WT TIGR4, Δ*ply*, or Δ*spxB* (B, E-F) at an MOI of 10. Data at hour 0 represent measurements taken just prior to infection. Data are displayed as the mean value of all replicates, comprised of technical triplicates from three independent iBEC differentiations. n = 9. One set of values was excluded from Δ*spxB* (Figure 4B) and mock (Figure 4C) due to a punctured transwell. Error bars represent SD. For TEER data, two-way ANOVA was used to determine significance between each condition across the various timepoints. *p* values are reported in the corresponding tables. For NaF data, one-way ANOVA was used to determine significance. Significance is defined as p < .05.

Next, we hypothesized that the loss of tight junctions in iBECs would correlate with the loss of barrier function. A common method for assessing barrier function is to measure transendothelial electrical resistance (TEER), with higher resistance indicating stronger barrier function and general tight junction integrity [95]. Previous work in other models shows that pneumococcal infection decreases the TEER of BECs [43,96]. The iBEC model has previously been utilized to assess changes in TEER during *S. agalactiae* and *N. meningitidis* infection [52,55]. To assess changes in TEER during pneumococcal infection, we cultured iBECs on 6.5 mm transwell inserts and infected the apical side with either mock or bacteria at an MOI of 10. To investigate whether pneumococcus could specifically decrease TEER, we compared TEER during infection with mock, TIGR4, and *L. lactis*, a nonpathogenic relative utilized previously as a negative control for *S. pneumoniae* [39]. As expected, we observed TEER in the mock group remain steady, whereas TEER in the TIGR4 infected group began to decrease around 5 hours P.I., and was completely diminished around 7 hours P.I (Figure 4(A)). While *L. lactis* did significantly decrease TEER compared to mock, it remained significantly higher than the TIGR4 condition and was above 1000 Ωxcm^2^ even at 8 hours P.I (Figure 4(A)). Significance between each condition across all timepoints is reported (Figure 4(A)). We likewise investigated barrier function during pneumococcal infection by assessing molecular permeability at 5 and 7 hrs P.I. Previous studies have utilized sodium fluorescein (NaF) to assess permeability of iBECs, and have shown that the loss of TEER typically correlates with an increase in permeability [55,63]. Likewise, we observed a significant increase in permeability at both 5 and 7 hours for *Spn*-infected cells, compared to mock (Figure 4(C,D)). While *L. lactis* significantly increases permeability at 7 hours (4D), there was no significant increase at 5 hours (Figure 4(C)), consistent with our TEER results. Next, we sought to identify specific pneumococcal factors important for inducing loss of TEER in iBECs. We hypothesized that the loss of TEER could be attributable to pneumococcal expression of cytotoxic compounds such as the toxin pneumolysin (Ply) or production of H_2_O_2_. Ply is the major pneumococcal toxin and has been shown to be cytotoxic to endothelial cells via its innate pore-forming activity [1,42]. Additionally, pneumococcus produces H_2_O_2_ via its pyruvate oxidase SpxB, which contributes to oxidative stress, DNA damage, and cell death [97]. Thus, we generated SpxB- and Ply-deficient mutants to study the impact of these two major pneumococcal virulence factors on iBECs. Interestingly, we found that while the SpxB-deficient mutant decreased TEER just as rapidly as the wildtype, the Ply-deficient mutant decreased TEER significantly slower than the wildtype (Figure 4(B)). Significance between each condition across all timepoints is reported (Figure 4(B)). When we repeated these conditions to assess NaF permeability, we observed that while neither mutant deviated from wildtype at 5 hours (Figure 4(E0), the Ply-deficient mutant increased permeability significantly less than wildtype at 7 hours (Figure 4(F)), consistent with TEER results. Interestingly, the SpxB-deficient mutant also seemed to increase permeability significantly less than wildtype at 7 hours (Figure 4(F)). This effect may be due to the fact that the SpxB mutant seemed to exhibit slower growth than either wildtype or the Ply mutant after 5 hours (Fig. S3B). However, the growth of the Ply mutant was indistinguishable from wildtype, supporting that the loss of Ply is sufficient to slow iBEC barrier disruption during infection (Fig S3B). Taken together, these results indicate that pneumococcus induces a loss of barrier function in iBECs and that this effect is partially mediated by the pneumococcal cytotoxin Ply.

### iBECs activate pro-inflammatory cytokines during pneumococcal infection

**Figure 5.**
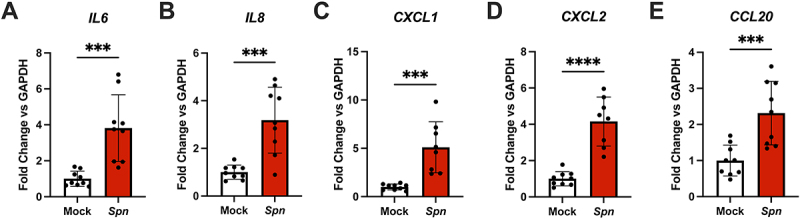
iBEC inflammatory cytokine expression during pneumococcal infection. iBECs were treated for 3.5 hours with mock or TIGR4 (*Spn)* at an MOI of 10. (A-E) RT-qPCR ΔΔCT values representing changes in expression of *IL6, IL8, CXCL1, CXCL2*, and *CCL20* during infection. *GAPDH* was used as the housekeeping gene. Data are displayed as the mean of all replicates, comprised of technical triplicates from three independent iBEC differentiations. *n* = 9. ROUT (Q = 1%) outlier test was applied to data sets and one outlier was removed from (C) and (D). Outlier values for (C) *Spn* and (D) *Spn* were 19.74 and 41.004, respectively. For (C) *Spn* and (D) *Spn*, *n* = 8. Error bars represent SD. Student’s t-test was utilized to determine significance between mock and *Spn*. *p < .05, **p < .01. ****p < .0001.

Next, we sought to determine whether iBECs responded to pneumococcal infection by initiating pro-inflammatory signaling pathways. Typically, BECs at the BBB are relatively immune-quiescent, meaning that they actively suppress the production of inflammatory chemokines and cytokines and limit the expression of leukocyte adhesion molecules [98]. Encounter with pneumococcus, however, typically results in the immune activation of the brain tissue and the BBB itself, characterized by a mass upregulation of inflammatory chemokines and cytokines [17,45,99]. iBECs maintain low basal levels of immune-related transcripts but may be activated during infection [50,52]. Thus, we infected iBEC monolayers with TIGR4 as previously described at an MOI of 10 for 3.5 hours, and then RNA was isolated and analyzed via RT-qPCR. We chose a panel of chemokines and cytokines comprised of IL-6, IL-8, CXCL-1, CXCL-2, and CCL20. The expression of each gene was significantly upregulated during pneumococcal infection (Figure 5(A-E)). This is consistent with previous work, and further supports iBECs as a valuable model for representing pneumococcus-BBB interaction.

## Discussion

Herein, we propose iPSC-derived brain-like endothelial cells, or iBECs, as a viable model for studying pneumococcal interaction with the BBB. Previous work has primarily relied on *in vivo* rodent or other animal models of the BBB to study pneumococcal meningitis [17,24,38,39,65,100,101]. *In vivo* models offer many irreplaceable advantages such as a holistic immune environment where the interplay between peripheral immune cells, the BBB, and the pathogen can be observed.

For example, *in vivo* models have been crucial for elucidating the role of reactive oxygen species (ROS) or reactive nitrogen species (RNS) and matrix metalloproteases (MMPs) to induce BBB-dysfunction. During pneumococcal meningitis, activated neutrophils and macrophages translocate to the CNS and contribute majorly to the production of ROS and RNS, both of which have been demonstrated to disrupt the BBB, induce neuronal damage, and contribute to long-term CNS complications [13,102]. Likewise, MMPs, which damage the BBB by degrading the endothelial basement membrane and disrupting tight junctions, experience significant upregulation during pneumococcal infection and are largely produced by activated phagocytes [103,104]. In addition to the role of phagocytes, compliment system activity also correlates with severity of pneumococcal meningitis, as compliment factor C5a has been shown to exacerbate brain damage in infected mice and humans [105], and compliment factor H deficiency was associated with increased mortality in infected mice and humans [106]. As various effectors of the innate immune response play a significant role in both protecting and disrupting the BBB during pneumococcal infection, study of pneumococcal meningitis in animal models remains essential.

Another key advantage of *in vivo* models is that they enable the study of neurological sequelae following pneumococcal meningitis. Even in high-resource countries, surviving adults frequently present with a variety of sequelae including focal deficits (11–36%), hearing loss (22–69%), seizures (31%), and cognitive impairment (32%) [8]. Murine models have been shown to be reliable tools for studying these neurological sequelae, as mice often present with hearing, memory, and spatial reasoning post-meningitis [107–109].

Despite the numerous benefits of *in vivo* models, they nevertheless feature key limitations which motivate the additional use of *in vitro* models. While a variety of animals may be experimentally infected with pneumococcus, the human nasopharynx is its only natural reservoir [110]. In this sense, pneumococcus is an obligate human pathogen, and its various virulence factors that enable colonization, horizontal transmission, and immune evasion are consequently adapted specifically to its human host. This is exemplified by the differential invasion patterns that various pneumococcal serotypes display between mice and humans – serotypes 14,19, and 23 are common clinical isolates in humans but are relatively avirulent in mice, whereas other serotypes are highly virulent in mice but insignificant for human disease [24]. Other studies note several general transcriptomic and proteomic differences between human and rodent BBBs – for instance, rodent BBBs feature far greater claudin-5 expression and p-glycoprotein function at the BBB than do humans [111]. As both proteins are crucial for maintaining BBB selectivity, their variation among species may lead to species-specific disruption of the BBB by pneumococcus. A practical limitation of *in vivo* models is that when infecting intranasally or intraperitoneally with pneumococcus to mimic the natural route of infection, around 50% of animals are reported to die of sepsis before developing meningitis [24]. Thus, even where biologically relevant, animal models present limitations of greater time, cost, and ethical concerns.

Applications of *in vitro* models are inherently far more limited in scope, as they typically represent an isolated cell type in a static environment. Nevertheless, they are not hindered by many of the practical limitations of *in vivo* models, and human *in vitro* models eliminate concerns of interspecies differences. For an obligate human pathogen such as pneumococcus, confirming *in vivo* findings in a human *in vitro* setting is particularly relevant. Both primary human cells and primary immortalized human cell lines have been utilized for the study of pneumococcal meningitis.

While human cultured primary cells or brain tissue samples are perhaps the most reliable *in vitro* models available [66,112], they require the surgical removal of tissue during a biopsy or autopsy, and thus availability is extremely limited [48]. Since primary cells tend to lose BBB characteristics upon removal from the brain microenvironment, they cannot be passaged repeatedly for continued usage, further restricting availability [18,21,48]. Additionally, primary human BECs are typically collected from tumor or epilepsy patients [21,113], which generates concerns that cells may display diseased phenotypes. Human primary BECs have been used importantly but infrequently to study pneumococcus at the BBB [42,65,90].

Prior work has typically utilized primary immortalized cell lines which are very accessible and highly scalable, as they may be passaged repeatedly without rapidly losing BBB-specific characteristics. The hCMEC/D3 cell line has rarely been used to study pneumococcal meningitis [45], while the hBMEC cell line immortalized via SV40 large T-antigen transfection has been frequently employed [17,37,39,43,44,114–117]. Both hBMECs and hCMEC/D3s exhibit several limitations for use as BBB models to study pneumococcus. While hBMECs express ZO-1, occludin, and VE-cadherin, they do not produce detectable levels of claudin-5 [47,52]. Claudin-5 forms *trans* interactions with claudin strands on adjacent cells and is the primary barrier-forming junction of the BBB [82]. Claudin-5 expression is crucial for the formation of an intact barrier [118], and its absence in hBMECs is a limitation for assessing the interaction between pneumococcus and the BBB. The hBMEC cells often do not produce detectable CD31, which is found ubiquitously in vascular endothelium [119]. As CD31 is a known receptor at the BBB for the pneumococcal pilus and CbpA [44,66], its lack or scarcity in hBMECs represents another limitation for modeling pneumococcal meningitis. hCMEC/D3s, while they express ZO-1 and VE-cadherin, along with CD31 and claudin-5, albeit in low abundance, display discontinuous expression of occludin [47,48,119]. Both hBMECs and hCMECs feature low TEER profiles, typically reported for both cell lines around 40 Ωxcm^2^ and as high as 200 Ωxcm^2^ in some systems [47,48,52,119]. TEER values as low as these offer little dynamic range for assessing changes in TEER induced by pneumococcal infection, as investigated in this study (Figure 4), and highlight another limitation of current human immortalized cell lines.

The current study illustrates many of the advantages that an iPSC-BEC model can offer compared to human immortalized models. iBECs reliably express VE-cadherin and CD31 and produce continuous ZO-1, occludin, and claudin-5 (Fig. S1). iBECs also reproduce physiologically relevant TEER values, measured around 3000 Ωxcm^2^ in this study (Figure 4), significantly improving on other *in vitro* models. Furthermore, unlike primary cells which typically originate from a diseased donor, the iPSCs from which the iBECs are derived are from a healthy donor. These improvements on current *in vitro* models couple with generic advantages over *in vivo* models such as iBECs’ availability, scalability, and human origin.

These strengths of the iPSC-BEC model, along with previous contributions of this model in studying a range of meningeal pathogens, clearly motivated our investigation of iBECs as a novel BBB model to study *Streptococcus pneumoniae*. Pneumococcus possesses an abundance of virulence factors that, while specialized to promote its survival and proliferation in its typical environment of the nasopharynx, also enable it to interact with and disrupt the BECs of the blood-brain barrier in cases of invasive pneumococcal disease. These virulence factors include adhesins like RrgA, CbpA (PspC), CbpE, CbpD and other choline-binding proteins, invasion-associated proteins like GAPDH and enolase, toxins or toxin-producing factors like SpxB and Ply, and the sialidase NanA [1]. To help determine whether pneumococcus interacts with iBECs via key virulence factors and host receptors, we selected its two major adhesins RrgA and CbpA. RrgA is located at the tip of a heterotrimeric pilus protein composed of RrgA, RrgB, and RrgC. All three genes encoding these proteins are located within the same operon, along with three sortase-encoding genes and a transcriptional regulator gene [120]. The pilus is only expressed in around 30% of carriage isolates, but appears to be nearly ubiquitous among strains able to cause meningitis [76,121]. While a previous report has shown that RrgB and RrgC are non-essential for pilus-mediated adherence [69], we utilized an isogenic mutant lacking the entire pilus locus. Choline-binding protein A (CbpA) is a surface-exposed protein of pneumococcus that functions in plasma protein binding and immune evasion, and also has inherent adhesive capabilities [122]. Previous work has shown that at the BBB, RrgA binds CD31 (Platelet/endothelial cell adhesion molecule 1 or PECAM-1) and the polymeric immunoglobulin receptor (pIgR) to facilitate adhesion, while CbpA binds pIgR to a lower extent [66]. As anticipated, knocking out either of these adhesins resulted in reduced adherence (Figure 1(K0), although our findings are inconclusive as to which receptors these adhesins interact with in iBECs.

We assessed pneumococcal disruption of the BBB first by examining different tight junction components essential for proper BBB function. Unlike current immortalized *in vitro* BBB models, iBECs produce claudin-5 and display continuous occludin expression [47,48,52], and thus iBECs constitute a valuable tool for investigating tight junction disruption at the BBB. Other work has observed that pneumococcal infection triggers the autosomal degradation of the tight junction protein occludin [85], and the loss of the tight junction scaffolding protein ZO-1 due to TGF-β-mediated downregulation [43]. We found both proteins to be downregulated in expression and abundance (Figure 2). Additionally, the analysis of junction images with JanaP also suggests the pneumococcus dysregulates the organization and continuity of these junctions (Figure 3). Although JanaP was helpful for analyzing ZO-1 and occludin, it was not useful for analyzing claudin-5 due to the level of background staining in these images. However, western blot analysis suggested that claudin-5 abundance was reduced after infection (Figure 2(D)). Although claudin-5 reduction due to pneumococcal infection has been observed previously in lung capillary cells [86], this has not been observed in a brain endothelial cell model. In support of these findings, we observed SNAIL1 to be overexpressed in infected iBECs (Figure 2H). SNAIL1 is a transcriptional regulator that suppresses the expression of tight junction components and has been observed to be upregulated in hBMECs infected with pneumococcus [43,87,89]. Vascular endothelial growth factor A (*VEGFA*) expression is also associated with the loss of tight junctions and was also upregulated during pneumococcal infection (Figure 2(I0). This is consistent with previous research which finds VEGF-A upregulated by pneumococcus [90,123]. Simultaneous VEGF-A and SNAIL1 upregulation are also observed during *E. coli* encounter with the BBB, further suggesting that these are conserved mechanisms of disruption [124]. What pneumococcal factors initiate VEGF-A and SNAIL1 activation are unknown and remain a subject for future research.

Since our data supported the disruption of iBEC tight junctions by pneumococcus, we assessed iBEC TEER and permeability during infection to determine whether pneumococcus would decrease the barrier function of iBECs. While TEER and molecular permeability are unable to elucidate the function of any specific barrier component, they offer a picture of barrier integrity as a whole, in which tight junctions play a major role. In multiple experiments, we report TEER values up to 3000 Ωxcm^2^ in iBECs (Figure 4(A,B)). This supports iBECs as a particularly valuable model for assessing changes in barrier function during infection. We hypothesized that the observed loss of tight junctions during pneumococcal infection would correspond to a loss of TEER and increase in permeability. As we observed a pneumococcus-specific induction of the loss of TEER and increase in NaF permeability in iBECs (Figure 4(A,C,D)), we hypothesized that pneumococcal Ply or SpxB might be responsible for this disruption. Ply is a pore-forming cytotoxin that drives inflammation and bacterial shedding and transmission in the nasopharynx. In the brain, it boosts neuroinflammation and endocytosis, and has been shown to damage the viability and barrier function of BECs *in vitro* [12,42]. SpxB is a pyruvate oxidase that produces H_2_O_2_ as a byproduct of its reaction, and thus its expression is potentially damaging to brain endothelial cells, where high levels of H_2_O_2_ induce apoptosis and the loss of tight junction integrity [114,125]. Additionally, SpxB supports the intracellular survival of pneumococcus in BECs by providing tolerance to endosomal acidification [114]. We observed that the loss of Ply limited the loss of iBEC barrier function as expected. Interestingly, the loss of SpxB did not rescue TEER but did seem to limit an increase in NaF permeability (Figure 4(B,E,F)), although this inconsistency may be explained by our finding that the SpxB mutant displayed a lag in growth relative to wildtype or the Ply mutant (Fig. S3B). Previous work shows that SpxB is important for virulence in colonization, pneumonia, and sepsis, but its impact on the barrier function of BECs has never been assessed [126]. However, it was previously shown in a murine model that while SpxB was important for nasopharyngeal colonization, replication in the lungs, and bloodstream invasion, invasion of the CSF was only CbpA- and Ply-dependent [32]. This finding aligns well with our findings, which suggest that Ply is more disruptive at the BBB than SpxB. Alternatively, since SpxB activity and H_2_O_2_ production are dependent on the growth phase of the bacteria and the availability of oxygen, our experimental conditions may have incidentally limited SpxB activity. Prior work in hBMECs has likewise demonstrated that the loss of Ply limits the loss of TEER induced by pneumococcus [42], supporting our finding in iBECs.

As anticipated, activation of *SNAI1* and *VEGFA* along with the disruption of tight junction proteins was accompanied by the upregulation of inflammatory transcripts. Interestingly, while a previous study reported that IL-6 was not upregulated in the iBEC model during *Streptococcus agalactiae* infection, and suggested that IL-6 might be produced by cells other than BECs *in vivo* [52], we found IL-6 to be significantly upregulated along with all other cytokines or chemokines assessed after pneumococcal infection (Figure 5A-E). Overall, we observe a robust inflammatory response from iBECs infected with pneumococcus, which provides additional justification for the loss of tight junctions and barrier integrity observed. Inflammatory signaling and cytokine production has been observed to dysregulate numerous aspects of the BBB including ABC transporter expression and tight junction presence [127]. Thus, while immune activation technically constitutes a protective response, it may further disrupt the function of the blood-brain barrier and thereby accelerate pneumococcus-induced disruption of the BBB.

Although the model used in this study consists only of iBECs, prior work has also demonstrated the successful generation of iPSC-derived astrocytes, pericytes, and neurons [128–130]. Moreover, iPSC-derived BECs, astrocytes, pericytes, and neurons may be cultured together to form a complete, isogenic neurovascular unit, which displays enhanced barrier properties relative to iBECs in monoculture [131]. After bypassing BECs, pneumococcus must cross the pericyte layer and astrocytic endfeet to access the brain parenchyma [10]. As previous work suggests that pericytes and astrocytes are protective of the BBB during pneumococcal infection [132], and that pneumococcus induces toxicity in astrocytes and neurons [133], this motivates further use of the iBEC model in coculture settings. Future work may utilize iBECs in conjunction with IPSC-derived astrocytes, pericytes, and neurons to investigate the interplay between these cell types and pneumococcus during meningitis.

## Supplementary Material

Supplemental Material

## Data Availability

The authors confirm that all data supporting the findings of this study are available in Figshare at http://doi.org/10.6084/m9.figshare.27045835 [134].

## Health and safety

All procedures were approved by the University of Alabama Institution Biosafety Committee for the use of pneumococcus on human cells. Procedures involving live pneumococcus were conducted in a Class II A2 biological safety cabinet, and live pneumococcus were inactivated prior to any molecular analysis. Pneumococcus can be transmitted via aerosol, therefore liquid cultures were kept at the lowest possible volume and solid media was preferred over liquid cultures.

## References

[1] Weiser JN, Ferreira DM, Paton JC. Streptococcus pneumoniae: transmission, colonization and invasion. Nat Rev Microbiol. 2018;16(6):355–21. doi: 10.1038/s41579-018-0001-8 29599457 PMC5949087

[2] Nakamura T, Cohen AL, Schwartz S, et al. The global landscape of pediatric bacterial meningitis data reported to the World Health Organization-coordinated Invasive Bacterial Vaccine-Preventable Disease Surveillance Network, 2014–2019. J Infect Dis. 2021;224(12 Suppl 2):S161–s173. doi: 10.1093/infdis/jiab217 34469555 PMC8409679

[3] Oordt-Speets AM, Bolijn R, van Hoorn RC, et al. Global etiology of bacterial meningitis: a systematic review and meta-analysis. PLOS ONE. 2018;13(6):e0198772. doi: 10.1371/journal.pone.0198772 29889859 PMC5995389

[4] Senders S, Klein NP, Lamberth E, et al. Safety and immunogenicity of a 20-valent pneumococcal conjugate vaccine in healthy infants in the United States. Pediatr Infect Dis J. 2021;40(10):944–951. doi: 10.1097/inf.0000000000003277 34525007 PMC8443440

[5] Grant LR, Meche A, McGrath L, et al. Risk of pneumococcal disease in US adults by age and risk profile. Open Forum Infect Dis. 2023;10(5):ofad192. doi: 10.1093/ofid/ofad192 37180598 PMC10167987

[6] Wang B, Lin W, Qian C, et al. Disease burden of meningitis caused by Streptococcus pneumoniae among under-fives in China: a systematic review and meta-analysis. Infect Dis Ther. 2023;12(11):2567–2580. doi: 10.1007/s40121-023-00878-y 37837523 PMC10651812

[7] Martín-Cerezuela M, Aseginolaza-Lizarazu M, Boronat-García P, et al. Severe community-acquired Streptococcus pneumoniae bacterial meningitis: clinical and prognostic picture from the intensive care unit. Crit Care. 2023;27(1):72. doi: 10.1186/s13054-023-04347-3 36823625 PMC9951455

[8] Lucas MJ, Brouwer MC, van de Beek D. Neurological sequelae of bacterial meningitis. J Infect. 2016;73(1):18–27. doi: 10.1016/j.jinf.2016.04.009 27105658

[9] Saha SK, Khan NZ, Ahmed AS, et al. Neurodevelopmental sequelae in pneumococcal meningitis cases in Bangladesh: a comprehensive follow-up study. Clin Infect Dis. 2009;48 Suppl 2(s2):S90–96. doi: 10.1086/596545 19191624

[10] Gil E, Wall E, Noursadeghi M, et al. Streptococcus pneumoniae meningitis and the CNS barriers. Front Cell Infect Microbiol. 2022;12:1106596. doi: 10.3389/fcimb.2022.1106596 36683708 PMC9845635

[11] Mohanty T, Fisher J, Bakochi A, et al. Neutrophil extracellular traps in the central nervous system hinder bacterial clearance during pneumococcal meningitis. Nat Commun. 2019;10(1):1667. doi: 10.1038/s41467-019-09040-0 30971685 PMC6458182

[12] Hupp S, Förtsch C, Graber F, et al. Pneumolysin boosts the neuroinflammatory response to Streptococcus pneumoniae through enhanced endocytosis. Nat Commun. 2022;13(1):5032. doi: 10.1038/s41467-022-32624-2 36028511 PMC9418233

[13] Mook-Kanamori BB, Geldhoff M, van der Poll T, et al. Pathogenesis and pathophysiology of pneumococcal meningitis. Clin Microbiol Rev. 2011;24(3):557–591. doi: 10.1128/cmr.00008-11 21734248 PMC3131058

[14] Weber JR, Tuomanen EI. Cellular damage in bacterial meningitis: an interplay of bacterial and host driven toxicity. J Neuroimmunol. 2007;184(1–2):45–52. doi: 10.1016/j.jneuroim.2006.11.016 17210186

[15] Audshasai T, Coles JA, Panagiotou S, et al. Streptococcus pneumoniae rapidly translocate from the nasopharynx through the cribriform plate to invade the outer meninges. MBio. 2022;13(4):e0102422. doi: 10.1128/mbio.01024-22 35924840 PMC9426477

[16] Farmen K, Tofiño-Vian M, Wellfelt K, et al. Spatio-temporal brain invasion pattern of Streptococcus pneumoniae and dynamic changes in the cellular environment in bacteremia-derived meningitis. Neurobiol Dis. 2024;195:106484. doi: 10.1016/j.nbd.2024.106484 38583642

[17] Iovino F, Orihuela CJ, Moorlag HE, et al. Interactions between blood-borne Streptococcus pneumoniae and the blood-brain barrier preceding meningitis. PLOS ONE. 2013;8(7):e68408. doi: 10.1371/journal.pone.0068408 23874613 PMC3713044

[18] Kim BJ, Shusta EV, Doran KS. Past and current perspectives in modeling bacteria and blood-brain barrier interactions. Front Microbiol. 2019;10:1336. doi: 10.3389/fmicb.2019.01336 31263460 PMC6585309

[19] Ayloo S, Gu C. Transcytosis at the blood-brain barrier. Curr Opin Neurobiol. 2019;57:32–38. doi: 10.1016/j.conb.2018.12.014 30708291 PMC6629499

[20] Espinal ER, Matthews T, Holder BM, et al. Group B streptococcus-induced macropinocytosis contributes to bacterial invasion of brain endothelial cells. Pathogens. 2022;11(4):474. doi: 10.3390/pathogens11040474 35456149 PMC9028350

[21] Lippmann ES, Azarin SM, Kay JE, et al. Derivation of blood-brain barrier endothelial cells from human pluripotent stem cells. Nat Biotechnol. 2012;30(8):783–791. doi: 10.1038/nbt.2247 22729031 PMC3467331

[22] Rössler K, Neuchrist C, Kitz K, et al. Expression of leucocyte adhesion molecules at the human blood-brain barrier (BBB). J Neurosci Res. 1992;31(2):365–374. doi: 10.1002/jnr.490310219 1374132

[23] Kadry H, Noorani B, Cucullo L. A blood–brain barrier overview on structure, function, impairment, and biomarkers of integrity. Fluids Barriers CNS. 2020;17(1):69. doi: 10.1186/s12987-020-00230-3 33208141 PMC7672931

[24] Chiavolini D, Pozzi G, Ricci S. Animal models of Streptococcus pneumoniae disease. Clin Microbiol Rev. 2008;21(4):666–685. doi: 10.1128/cmr.00012-08 18854486 PMC2570153

[25] Gehre F, Leib SL, Grandgirard D, et al. Essential role of choline for pneumococcal virulence in an experimental model of meningitis. J Intern Med. 2008;264(2):143–154. doi: 10.1111/j.1365-2796.2008.01930.x 18331292

[26] Saukkonen K, Sande S, Cioffe C, et al. The role of cytokines in the generation of inflammation and tissue damage in experimental gram-positive meningitis. J Exp Med. 1990;171(2):439–448. doi: 10.1084/jem.171.2.439 2406363 PMC2187712

[27] Suntur BM, Yurtseven T, Sipahi OR, et al. Rifampicin+ceftriaxone versus vancomycin+ceftriaxone in the treatment of penicillin- and cephalosporin-resistant pneumococcal meningitis in an experimental rabbit model. Int J Antimicrob Agents. 2005;26(3):258–260. doi: 10.1016/j.ijantimicag.2005.06.010 16099624

[28] Tuomanen E, Liu H, Hengstler B, et al. The induction of meningeal inflammation by components of the pneumococcal cell wall. J Infect Dis. 1985;151(5):859–868. doi: 10.1093/infdis/151.5.859 3989321

[29] Xu D, Lian D, Wu J, et al. Brain-derived neurotrophic factor reduces inflammation and hippocampal apoptosis in experimental Streptococcus pneumoniae meningitis. J Neuroinflammation. 2017;14(1):156. doi: 10.1186/s12974-017-0930-6 28778220 PMC5545027

[30] Chen A, Mann B, Gao G, et al. Multivalent pneumococcal protein vaccines comprising pneumolysoid with epitopes/fragments of CbpA and/or PspA elicit strong and broad protection. Clin Vaccine Immunol. 2015;22(10):1079–1089. doi: 10.1128/cvi.00293-15 26245351 PMC4580740

[31] Marra A, Brigham D. Streptococcus pneumoniae causes experimental meningitis following intranasal and otitis media infections via a nonhematogenous route. Infect Immun. 2001;69(12):7318–7325. doi: 10.1128/iai.69.12.7318-7325.2001 11705903 PMC98817

[32] Orihuela CJ, Gao G, Francis KP, et al. Tissue-specific contributions of pneumococcal virulence factors to pathogenesis. J Infect Dis. 2004;190(9):1661–1669. doi: 10.1086/424596 15478073

[33] Orihuela CJ, Gao G, McGee M, et al. Organ-specific models of Streptococcus pneumoniae disease. Scand J Infect Dis. 2003;35(9):647–652. doi: 10.1080/00365540310015854 14620149

[34] Rodriguez AF, Kaplan SL, Hawkins EP, et al. Hematogenous pneumococcal meningitis in the infant rat: description of a model. J Infect Dis. 1991;164(6):1207–1209. doi: 10.1093/infdis/164.6.1207 1835472

[35] Saladino RA, Stack AM, Fleisher GR, et al. Development of a model of low-inoculum Streptococcus pneumoniae intrapulmonary infection in infant rats. Infect Immun. 1997;65(11):4701–4704. doi: 10.1128/iai.65.11.4701-4704.1997 9353053 PMC175674

[36] Zwijnenburg PJ, van der Poll T, Florquin S, et al. Experimental pneumococcal meningitis in mice: a model of intranasal infection. J Infect Dis. 2001;183(7):1143–1146. doi: 10.1086/319271 11237845

[37] Banerjee A, Van Sorge NM, Sheen TR, et al. Activation of brain endothelium by pneumococcal neuraminidase NanA promotes bacterial internalization. Cell Microbiol. 2010;12(11):1576–1588. doi: 10.1111/j.1462-5822.2010.01490.x 20557315 PMC2943548

[38] Tan TQ, Smith CW, Hawkins EP, et al. Hematogenous bacterial meningitis in an intercellular adhesion molecule-1-deficient infant mouse model. J Infect Dis. 1995;171(2):342–349. doi: 10.1093/infdis/171.2.342 7844370

[39] Uchiyama S, Carlin AF, Khosravi A, et al. The surface-anchored NanA protein promotes pneumococcal brain endothelial cell invasion. J Exp Med. 2009;206(9):1845–1852. doi: 10.1084/jem.20090386 19687228 PMC2737157

[40] McCombs JE, Kohler JJ. Pneumococcal neuraminidase substrates identified through comparative proteomics enabled by chemoselective labeling. Bioconjug Chem. 2016;27(4):1013–1022. doi: 10.1021/acs.bioconjchem.6b00050 26954852 PMC4838540

[41] Rahman NA, Sharudin A, Diah S, et al. Serotyping of Brunei pneumococcal clinical strains and the investigation of their capability to adhere and invade a brain endothelium model. Microb Pathog. 2017;110:352–358. doi: 10.1016/j.micpath.2017.07.021 28711510

[42] Zysk G, Schneider-Wald BK, Hwang JH, et al. Pneumolysin is the main inducer of cytotoxicity to brain microvascular endothelial cells caused by Streptococcus pneumoniae. Infect Immun. 2001;69(2):845–852. doi: 10.1128/iai.69.2.845-852.2001 11159977 PMC97961

[43] Gratz N, Loh LN, Mann B, et al. Pneumococcal neuraminidase activates TGF-β signalling. Microbiology (Reading). 2017;163(8):1198–1207. doi: 10.1099/mic.0.000511 28749326 PMC5817201

[44] Iovino F, Molema G, Bijlsma JJ. Platelet endothelial cell adhesion molecule-1, a putative receptor for the adhesion of Streptococcus pneumoniae to the vascular endothelium of the blood-brain barrier. Infect Immun. 2014;82(9):3555–3566. doi: 10.1128/iai.00046-14 24914219 PMC4187830

[45] Jiménez-Munguía I, Tomečková Z, Mochnáčová E, et al. Transcriptomic analysis of human brain microvascular endothelial cells exposed to laminin binding protein (adhesion lipoprotein) and Streptococcus pneumoniae. Sci Rep. 2021;11(1):7970. doi: 10.1038/s41598-021-87021-4 33846455 PMC8041795

[46] Lippmann ES, Al-Ahmad A, Azarin SM, et al. A retinoic acid-enhanced, multicellular human blood-brain barrier model derived from stem cell sources. Sci Rep. 2014;4(1):4160. doi: 10.1038/srep04160 24561821 PMC3932448

[47] Eigenmann DE, Xue G, Kim KS, et al. Comparative study of four immortalized human brain capillary endothelial cell lines, hCMEC/D3, hBMEC, TY10, and BB19, and optimization of culture conditions, for an in vitro blood-brain barrier model for drug permeability studies. Fluids Barriers CNS. 2013;10(1):33. doi: 10.1186/2045-8118-10-33 24262108 PMC4176484

[48] Helms HC, Abbott NJ, Burek M, et al. In vitro models of the blood-brain barrier: an overview of commonly used brain endothelial cell culture models and guidelines for their use. J Cereb Blood Flow Metab. 2016;36(5):862–890. doi: 10.1177/0271678x16630991 26868179 PMC4853841

[49] Alimonti JB, Ribecco-Lutkiewicz M, Sodja C, et al. Zika virus crosses an in vitro human blood brain barrier model. Fluids Barriers CNS. 2018;15(1):15. doi: 10.1186/s12987-018-0100-y 29759080 PMC5952854

[50] Endres LM, Hathcock SF, Schubert-Unkmeir A, et al. Neisseria meningitidis infection of induced pluripotent stem-cell derived brain endothelial cells. J Vis Exp. 2020;(161). doi: 10.3791/61400 32744533

[51] Espinal ER, Sharp SJ, Kim BJ. Induced pluripotent stem cell (iPSC)-derived endothelial cells to study bacterial-brain endothelial cell interactions. Methods Mol Biol. 2022;2492:73–101. doi: 10.1007/978-1-0716-2289-6_4 35733039

[52] Kim BJ, Bee OB, McDonagh MA, et al. Modeling group B streptococcus and blood-brain barrier interaction by using induced pluripotent stem cell-derived brain endothelial cells. mSphere. 2017;2(6). doi: 10.1128/mSphere.00398-17 PMC5663983 29104935

[53] Kim BJ, McDonagh MA, Deng L, et al. Streptococcus agalactiae disrupts P-glycoprotein function in brain endothelial cells. Fluids Barriers CNS. 2019;16(1):26. doi: 10.1186/s12987-019-0146-5 31434575 PMC6704684

[54] Mamana J, Humber GM, Espinal ER, et al. Coxsackievirus B3 infects and disrupts human induced-pluripotent stem cell derived brain-like endothelial cells. Front Cell Infect Microbiol. 2023;13:1171275. doi: 10.3389/fcimb.2023.1171275 37139492 PMC10149843

[55] Martins Gomes SF, Westermann AJ, Sauerwein T, et al. Induced pluripotent stem cell-derived brain endothelial cells as a cellular model to study Neisseria meningitidis infection. Front Microbiol. 2019;10:1181. doi: 10.3389/fmicb.2019.01181 31191497 PMC6548865

[56] Vollmuth N, Sin J, Kim BJ. Host-microbe interactions at the blood-brain barrier through the lens of induced pluripotent stem cell-derived brain-like endothelial cells. MBio. 2024;15(2):e0286223. doi: 10.1128/mbio.02862-23 38193670 PMC10865987

[57] Yamada S, Hashita T, Yanagida S, et al. SARS-CoV-2 causes dysfunction in human iPSC-derived brain microvascular endothelial cells potentially by modulating the Wnt signaling pathway. Fluids Barriers CNS. 2024;21(1):32. doi: 10.1186/s12987-024-00533-9 38584257 PMC11000354

[58] Hu Y, Park N, Seo KS, et al. Pneumococcal surface adhesion A protein (PsaA) interacts with human annexin A2 on airway epithelial cells. Virulence. 2021;12(1):1841–1854. doi: 10.1080/21505594.2021.1947176 34233589 PMC8274441

[59] Tettelin H, Nelson KE, Paulsen IT, et al. Complete genome sequence of a virulent isolate of Streptococcus pneumoniae. Science. 2001;293(5529):498–506. doi: 10.1126/science.1061217 11463916

[60] Aaberge IS, Eng J, Lermark G, et al. Virulence of Streptococcus pneumoniae in mice: a standardized method for preparation and frozen storage of the experimental bacterial inoculum. Microb Pathog. 1995;18(2):141–152. doi: 10.1016/s0882-4010(95)90125-6 7643743

[61] Bryant JC, Dabbs RC, Oswalt KL, et al. Pyruvate oxidase of Streptococcus pneumoniae contributes to pneumolysin release. BMC Microbiol. 2016;16(1):271. doi: 10.1186/s12866-016-0881-6 27829373 PMC5103497

[62] Thornton JA. Splicing by overlap extension PCR to obtain hybrid DNA products. Methods Mol Biol. 2016;1373:43–49. doi: 10.1007/7651_2014_182 25646606

[63] Stebbins MJ, Wilson HK, Canfield SG, et al. Differentiation and characterization of human pluripotent stem cell-derived brain microvascular endothelial cells. Methods. 2016;101:93–102. doi: 10.1016/j.ymeth.2015.10.016 26518252 PMC4848177

[64] Gray KM, Katz DB, Brown EG, et al. Quantitative phenotyping of cell-cell junctions to evaluate ZO-1 presentation in brain endothelial cells. Ann Biomed Eng. 2019;47(7):1675–1687. doi: 10.1007/s10439-019-02266-5 30993538

[65] Orihuela CJ, Mahdavi J, Thornton J, et al. Laminin receptor initiates bacterial contact with the blood brain barrier in experimental meningitis models. J Clin Invest. 2009;119(6):1638–1646. doi: 10.1172/jci36759 19436113 PMC2689107

[66] Iovino F, Engelen-Lee JY, Brouwer M, et al. pIgR and PECAM-1 bind to pneumococcal adhesins RrgA and PspC mediating bacterial brain invasion. J Exp Med. 2017;214(6):1619–1630. doi: 10.1084/jem.20161668 28515075 PMC5461002

[67] Paton JC, Trappetti C, Fischetti VA, et al. *Streptococcus pneumoniae* capsular polysaccharide. Microbiol Spectr. 2019;7(2):10–128. doi: 10.1128/microbiolspec.gpp3-0019-2018 PMC11590643 30977464

[68] Weiser JN, Bae D, Fasching C, et al. Antibody-enhanced pneumococcal adherence requires IgA1 protease. Proc Natl Acad Sci USA. 2003;100(7):4215–4220. doi: 10.1073/pnas.0637469100 12642661 PMC153073

[69] Nelson AL, Ries J, Bagnoli F, et al. Rrga is a pilus-associated adhesin in Streptococcus pneumoniae. Mol Microbiol. 2007;66(2):329–340. doi: 10.1111/j.1365-2958.2007.05908.x 17850254 PMC2170534

[70] Rosenow C, Ryan P, Weiser JN, et al. Contribution of novel choline-binding proteins to adherence, colonization and immunogenicity of Streptococcus pneumoniae. Mol Microbiol. 1997;25(5):819–829. doi: 10.1111/j.1365-2958.1997.mmi494.x 9364908

[71] Hammerschmidt S, Talay SR, Brandtzaeg P, et al. SpsA, a novel pneumococcal surface protein with specific binding to secretory immunoglobulin A and secretory component. Mol Microbiol. 1997;25(6):1113–1124. doi: 10.1046/j.1365-2958.1997.5391899.x 9350867

[72] Elm C, Braathen R, Bergmann S, et al. Ectodomains 3 and 4 of human polymeric immunoglobulin receptor (hpIgr) mediate invasion of Streptococcus pneumoniae into the epithelium. J Biol Chem. 2004;279(8):6296–6304. doi: 10.1074/jbc.M310528200 14660617

[73] Zhang JR, Mostov KE, Lamm ME, et al. The polymeric immunoglobulin receptor translocates pneumococci across human nasopharyngeal epithelial cells. Cell. 2000;102(6):827–837. doi: 10.1016/s0092-8674(00)00071-4 11030626

[74] Barocchi MA, Ries J, Zogaj X, et al. A pneumococcal pilus influences virulence and host inflammatory responses. Proc Natl Acad Sci USA. 2006;103(8):2857–2862. doi: 10.1073/pnas.0511017103 16481624 PMC1368962

[75] LeMieux J, Hava DL, Basset A, et al. Rrga and Rrgb are components of a multisubunit pilus encoded by the Streptococcus pneumoniae rlrA pathogenicity islet. Infect Immun. 2006;74(4):2453–2456. doi: 10.1128/iai.74.4.2453-2456.2006 16552078 PMC1418942

[76] Iovino F, Hammarlöf DL, Garriss G, et al. Pneumococcal meningitis is promoted by single cocci expressing pilus adhesin RrgA. J Clin Invest. 2016;126(8):2821–2826. doi: 10.1172/jci84705 27348589 PMC4966305

[77] Becke TD, Ness S, Gürster R, et al. Single molecule force spectroscopy reveals two-domain binding mode of pilus-1 tip protein RrgA of Streptococcus pneumoniae to fibronectin. ACS Nano. 2018;12(1):549–558. doi: 10.1021/acsnano.7b07247 29298375

[78] Becke TD, Ness S, Kaufmann BK, et al. Pilus-1 backbone protein RrgB of Streptococcus pneumoniae binds collagen I in a force-dependent way. ACS Nano. 2019;13(6):7155–7165. doi: 10.1021/acsnano.9b02587 31184856

[79] Izoré T, Contreras-Martel C, El Mortaji L, et al. Structural basis of host cell recognition by the pilus adhesin from Streptococcus pneumoniae. Structure. 2010;18(1):106–115. doi: 10.1016/j.str.2009.10.019 20152157

[80] Voss S, Hallström T, Saleh M, et al. The choline-binding protein PspC of Streptococcus pneumoniae interacts with the C-terminal heparin-binding domain of vitronectin. J Biol Chem. 2013;288(22):15614–15627. doi: 10.1074/jbc.M112.443507 23603906 PMC3668722

[81] Dithmer S, Blasig IE, Fraser PA, et al. The basic requirement of tight junction proteins in blood-brain barrier function and their role in pathologies. Int J Mol Sci. 2024;25(11):5601. doi: 10.3390/ijms25115601 38891789 PMC11172262

[82] Hashimoto Y, Campbell M. Tight junction modulation at the blood-brain barrier: current and future perspectives. Biochim Biophys Acta Biomembr. 2020;1862(9):183298. doi: 10.1016/j.bbamem.2020.183298 32353377

[83] Attali C, Durmort C, Vernet T, et al. The interaction of Streptococcus pneumoniae with plasmin mediates transmigration across endothelial and epithelial monolayers by intercellular junction cleavage. Infect Immun. 2008;76(11):5350–5356. doi: 10.1128/iai.00184-08 18725422 PMC2573366

[84] Clarke TB, Francella N, Huegel A, et al. Invasive bacterial pathogens exploit TLR-mediated downregulation of tight junction components to facilitate translocation across the epithelium. Cell Host Microbe. 2011;9(5):404–414. doi: 10.1016/j.chom.2011.04.012 21575911 PMC4536975

[85] Cui L, Yang R, Huo D, et al. Streptococcus pneumoniae extracellular vesicles aggravate alveolar epithelial barrier disruption via autophagic degradation of OCLN (occludin). Autophagy. 2024;20(7):1–20. doi: 10.1080/15548627.2024.2330043 38497494 PMC11210924

[86] Peter A, Fatykhova D, Kershaw O, et al. Localization and pneumococcal alteration of junction proteins in the human alveolar-capillary compartment. Histochem Cell Biol. 2017;147(6):707–719. doi: 10.1007/s00418-017-1551-y 28247028

[87] Liu S, Shen Y, Feng T, et al. Effects of silencing Snail1 gene on the expression of tight junction proteins and the migration ability of Hep-2 cells. Sheng Wu Yi Xue Gong Cheng Xue Za Zhi. 2017;34(4):591–596. doi: 10.7507/1001-5515.201702005 29745557 PMC9935324

[88] Vincent T, Neve EP, Johnson JR, et al. A SNAIL1-SMAD3/4 transcriptional repressor complex promotes TGF-beta mediated epithelial-mesenchymal transition. Nat Cell Biol. 2009;11(8):943–950. doi: 10.1038/ncb1905 19597490 PMC3769970

[89] Kim BJ, Hancock BM, Bermudez A, et al. Bacterial induction of Snail1 contributes to blood-brain barrier disruption. J Clin Invest. 2015;125(6):2473–2483. doi: 10.1172/jci74159 25961453 PMC4497739

[90] Devraj G, Guérit S, Seele J, et al. HIF-1α is involved in blood-brain barrier dysfunction and paracellular migration of bacteria in pneumococcal meningitis. Acta Neuropathol. 2020;140(2):183–208. doi: 10.1007/s00401-020-02174-2 32529267 PMC7360668

[91] Lan G, Wang P, Chan RB, et al. Astrocytic VEGFA: an essential mediator in blood-brain-barrier disruption in Parkinson’s disease. Glia. 2022;70(2):337–353. doi: 10.1002/glia.24109 34713920

[92] Wu M, Gong Y, Jiang L, et al. Vegf regulates the blood‑brain barrier through MMP‑9 in a rat model of traumatic brain injury. Exp Ther Med. 2022;24(6):728. doi: 10.3892/etm.2022.11664 36382093 PMC9634340

[93] Yang R, Chen J, Qu X, et al. Interleukin-22 contributes to blood-brain barrier disruption via STAT3/VEGFA activation in Escherichia coli meningitis. ACS Infect Dis. 2024;10(3):988–999. doi: 10.1021/acsinfecdis.3c00668 38317607 PMC10928716

[94] Gray KM, Jung JW, Inglut CT, et al. Quantitatively relating brain endothelial cell-cell junction phenotype to global and local barrier properties under varied culture conditions via the junction analyzer program. Fluids Barriers CNS. 2020;17(1):16. doi: 10.1186/s12987-020-0177-y 32046757 PMC7014765

[95] Brandl S, Reindl M. Blood-brain barrier breakdown in neuroinflammation: current in vitro models. Int J Mol Sci. 2023;24(16):12699. doi: 10.3390/ijms241612699 37628879 PMC10454051

[96] Yau B, Hunt NH, Mitchell AJ, et al. Blood‒brain barrier pathology and CNS outcomes in Streptococcus pneumoniae meningitis. Int J Mol Sci. 2018;19(11):3555. doi: 10.3390/ijms19113555 30423890 PMC6275034

[97] Rai P, Parrish M, Tay IJ, et al. Streptococcus pneumoniae secretes hydrogen peroxide leading to DNA damage and apoptosis in lung cells. Proc Natl Acad Sci USA. 2015;112(26):E3421–3430. doi: 10.1073/pnas.1424144112 26080406 PMC4491788

[98] van Doorn R, Lopes Pinheiro MA, Kooij G, et al. Sphingosine 1-phosphate receptor 5 mediates the immune quiescence of the human brain endothelial barrier. J Neuroinflammation. 2012;9:133. doi: 10.1186/1742-2094-9-133 22715976 PMC3425155

[99] Loughran AJ, Orihuela CJ, Tuomanen EI. Streptococcus pneumoniae: invasion and inflammation. Microbiol Spectr. 2019;7(2). doi: 10.1128/microbiolspec.GPP3-0004-2018 PMC6422050 30873934

[100] Täuber MG, Sande MA. Pathogenesis of bacterial meningitis: contributions by experimental models in rabbits. Infection. 1984;12 Suppl 1(S1):S3–10. doi: 10.1007/bf01641732 6397452

[101] Yau B, Too LK, Ball HJ, et al. Tigr4 strain causes more severe disease than Wu2 strain in a mouse model of Streptococcus pneumoniae meningitis: a common pathogenic role for interferon-γ. Microbes Infect. 2017;19(7–8):413–421. doi: 10.1016/j.micinf.2017.04.002 28438705

[102] Klein M, Koedel U, Pfister HW. Oxidative stress in pneumococcal meningitis: a future target for adjunctive therapy? Prog Neurobiol. 2006;80(6):269–280. doi: 10.1016/j.pneurobio.2006.11.008 17215069

[103] Leib SL, Leppert D, Clements J, et al. Matrix metalloproteinases contribute to brain damage in experimental pneumococcal meningitis. Infect Immun. 2000;68(2):615–620. doi: 10.1128/iai.68.2.615-620.2000 10639424 PMC97183

[104] Yang Y, Estrada EY, Thompson JF, et al. Matrix metalloproteinase-mediated disruption of tight junction proteins in cerebral vessels is reversed by synthetic matrix metalloproteinase inhibitor in focal ischemia in rat. J Cereb Blood Flow Metab. 2007;27(4):697–709. doi: 10.1038/sj.jcbfm.9600375 16850029

[105] Woehrl B, Brouwer MC, Murr C, et al. Complement component 5 contributes to poor disease outcome in humans and mice with pneumococcal meningitis. J Clin Invest. 2011;121(10):3943–3953. doi: 10.1172/jci57522 21926466 PMC3195471

[106] Kasanmoentalib ES, Valls Serón M, Engelen-Lee JY, et al. Complement factor H contributes to mortality in humans and mice with bacterial meningitis. J neuroinflammation. 2019;16(1):279. doi: 10.1186/s12974-019-1675-1 31883521 PMC6935240

[107] Hirose K, Li SZ, Gill R, et al. Pneumococcal meningitis induces hearing loss and cochlear ossification modulated by chemokine receptors CX3CR1 and CCR2. J Assoc Res Otolaryngol. 2024;25(2):179–199. doi: 10.1007/s10162-024-00935-4 38472515 PMC11018586

[108] Klein M, Schmidt C, Kastenbauer S, et al. MyD88-dependent immune response contributes to hearing loss in experimental pneumococcal meningitis. J Infect Dis. 2007;195(8):1189–1193. doi: 10.1086/512859 17357057

[109] Wellmer A, Noeske C, Gerber J, et al. Spatial memory and learning deficits after experimental pneumococcal meningitis in mice. Neurosci Lett. 2000;296(2–3):137–140. doi: 10.1016/s0304-3940(00)01645-1 11109000

[110] Lu L, Ma Z, Jokiranta TS, et al. Species-specific interaction of Streptococcus pneumoniae with human complement factor H. J Immunol. 2008;181(10):7138–7146. doi: 10.4049/jimmunol.181.10.7138 18981135 PMC2587499

[111] Aday S, Cecchelli R, Hallier-Vanuxeem D, et al. Stem cell-based human blood-brain barrier models for drug discovery and delivery. Trends Biotechnol. 2016;34(5):382–393. doi: 10.1016/j.tibtech.2016.01.001 26838094

[112] Bernas MJ, Cardoso FL, Daley SK, et al. Establishment of primary cultures of human brain microvascular endothelial cells to provide an in vitro cellular model of the blood-brain barrier. Nat Protoc. 2010;5(7):1265–1272. doi: 10.1038/nprot.2010.76 20595955 PMC3109429

[113] Cecchelli R, Berezowski V, Lundquist S, et al. Modelling of the blood-brain barrier in drug discovery and development. Nat Rev Drug Discov. 2007;6(8):650–661. doi: 10.1038/nrd2368 17667956

[114] Anil A, Apte S, Joseph J, et al. Pyruvate oxidase as a key determinant of pneumococcal viability during transcytosis across brain endothelium. J Bacteriol. 2021;203(24):e0043921. doi: 10.1128/jb.00439-21 34606370 PMC8604078

[115] Cheng Z, Zheng Y, Yang W, et al. Pathogenic bacteria exploit transferrin receptor transcytosis to penetrate the blood-brain barrier. Proc Natl Acad Sci USA. 2023;120(39):e2307899120. doi: 10.1073/pnas.2307899120 37733740 PMC10523449

[116] Nizet V, Kim KS, Stins M, et al. Invasion of brain microvascular endothelial cells by group B streptococci. Infect Immun. 1997;65(12):5074–5081. doi: 10.1128/iai.65.12.5074-5081.1997 9393798 PMC175731

[117] Stins MF, Badger J, Sik Kim K. Bacterial invasion and transcytosis in transfected human brain microvascular endothelial cells. Microb Pathog. 2001;30(1):19–28. doi: 10.1006/mpat.2000.0406 11162182

[118] Greene C, Hanley N, Campbell M. Claudin-5: gatekeeper of neurological function. Fluids Barriers CNS. 2019;16(1):3. doi: 10.1186/s12987-019-0123-z 30691500 PMC6350359

[119] Taggi V, Schäfer AM, Dolce A, et al. A face-to-face comparison of the BBB cell models hCMEC/D3 and hBMEC for their applicability to adenoviral expression of transporters. J Neurochem. 2024;168(9):2611–2620. doi: 10.1111/jnc.16125 38735840

[120] Miao C, Cui Y, Yan Z, et al. Pilus of Streptococcus pneumoniae: structure, function and vaccine potential. Front Cell Infect Microbiol. 2023;13:1270848. doi: 10.3389/fcimb.2023.1270848 37799336 PMC10548224

[121] Iovino F, Nannapaneni P, Henriques-Normark B, et al. The impact of the ancillary pilus-1 protein RrgA of Streptococcus pneumoniae on colonization and disease. Mol Microbiol. 2020;113(3):650–658. doi: 10.1111/mmi.14451 32185835

[122] Vilhena C, Du S, Battista M, et al. The choline-binding proteins PspA, PspC, and LytA of Streptococcus pneumoniae and their interaction with human endothelial and red blood cells. Infect Immun. 2023;91(9):e0015423. doi: 10.1128/iai.00154-23 37551971 PMC10501214

[123] van Der Flier M, Coenjaerts F, Kimpen JL, et al. Streptococcus pneumoniae induces secretion of vascular endothelial growth factor by human neutrophils. Infect Immun. 2000;68(8):4792–4794. doi: 10.1128/iai.68.8.4792-4794.2000 10899891 PMC98440

[124] Yang R, Liu W, Miao L, et al. Induction of VEGFA and Snail-1 by meningitic Escherichia coli mediates disruption of the blood-brain barrier. Oncotarget. 2016;7(39):63839–63855. doi: 10.18632/oncotarget.11696 27588479 PMC5325408

[125] Anasooya Shaji C, Robinson BD, Yeager A, et al. The tri-phasic role of hydrogen peroxide in blood-brain barrier endothelial cells. Sci Rep. 2019;9(1):133. doi: 10.1038/s41598-018-36769-3 30644421 PMC6333800

[126] Spellerberg B, Cundell DR, Sandros J, et al. Pyruvate oxidase, as a determinant of virulence in Streptococcus pneumoniae. Mol Microbiol. 1996;19(4):803–813. doi: 10.1046/j.1365-2958.1996.425954.x 8820650

[127] Zhao Y, Gan L, Ren L, et al. Factors influencing the blood-brain barrier permeability. Brain Res. 2022;1788:147937. doi: 10.1016/j.brainres.2022.147937 35568085

[128] Canfield SG, Stebbins MJ, Morales BS, et al. An isogenic blood-brain barrier model comprising brain endothelial cells, astrocytes, and neurons derived from human induced pluripotent stem cells. J Neurochem. 2017;140(6):874–888. doi: 10.1111/jnc.13923 27935037 PMC5339046

[129] Stebbins MJ, Gastfriend BD, Canfield SG, et al. Human pluripotent stem cell-derived brain pericyte-like cells induce blood-brain barrier properties. Sci Adv. 2019;5(3):eaau7375. doi: 10.1126/sciadv.aau7375 30891496 PMC6415958

[130] Tcw J, Wang M, Pimenova AA, et al. An efficient platform for astrocyte differentiation from human induced pluripotent stem cells. STEM Cell Rep. 2017;9(2):600–614. doi: 10.1016/j.stemcr.2017.06.018 PMC5550034 28757165

[131] Canfield SG, Stebbins MJ, Faubion MG, et al. An isogenic neurovascular unit model comprised of human induced pluripotent stem cell-derived brain microvascular endothelial cells, pericytes, astrocytes, and neurons. Fluids Barriers CNS. 2019;16(1):25. doi: 10.1186/s12987-019-0145-6 31387594 PMC6685239

[132] Teske NC, Dyckhoff-Shen S, Beckenbauer P, et al. Pericytes are protective in experimental pneumococcal meningitis through regulating leukocyte infiltration and blood-brain barrier function. J Neuroinflammation. 2023;20(1):267. doi: 10.1186/s12974-023-02938-z 37978545 PMC10655320

[133] Kim YS, Kennedy S, Täuber MG. Toxicity of Streptococcus pneumoniae in neurons, astrocytes, and microglia in vitro. J Infect Dis. 1995;171(5):1363–1368. doi: 10.1093/infdis/171.5.1363 7751718

[134] Mauser HD. Stem cell-derived brain-like endothelial cells to interrogate Streptococcus pneumoniae interaction with brain endothelium. (Version 2). 2025. doi: 10.6084/m9.figshare.27045835 PMC12498544 41042679

