# Supplementary material for: Stem cell-derived brain-like endothelial cells to interrogate Streptococcus pneumoniae interaction with brain endothelium
Source: Virulence. 2025 Oct 3;16(1):2564281. doi: 10.1080/21505594.2025.2564281 (PMC12498544; doi:10.1080/21505594.2025.2564281)
Supplement: Supplemental Material [file KVIR_A_2564281_SM3956.zip › QVIR Supplement Table 1.docx]

Supplement Table 1: Antibodies used in this study

| **Antibody** | **Dilution** | **Product ID** |
| --- | --- | --- |
| CD31 Monoclonal Antibody | 1:25 | Thermo cat#MA5-13188 |
| Claudin 5 Monoclonal Antibody | 1:50 | Thermo cat#35-2500 |
| GAPDH Monoclonal Antibody | 1:25000 | Thermo cat#AM4300 |
| GLUT1 Monoclonal Antibody | 1:100 | Thermo cat#MA5-11315 |
| Occludin Monoclonal Antibody | 1:200 | Thermo cat#33-1500 |
| p53 Monoclonal Antibody | 1:500 | Thermo cat#MA5-12557 |
| P-glycoprotein Monoclonal Antibody | 1:25 | Thermo cat#MA5-13854 |
| PIGR Monoclonal Antibody | 1:200 | Thermo cat#MA5-48571 |
| S. pneumoniae Polyclonal Antibody | 1:200 | Thermo cat#PA1-7259 |
| VE-cadherin Antibody | 1:25 | Scbt cat#sc-52751 |
| ZO-1 Monoclonal Antibody | 1:100 | Thermo cat#ZO-1-1A12 |
| Goat anti-mouse Alexa Fluor™ 488 secondary | 1:200 | Thermo cat#A11001 |
| Goat anti-mouse Texas Red-X secondary | 1:1000 | Thermo cat#T6390 |
| Goat anti-rabbit Alexa Fluor™ 488 secondary | 1:200 | Thermo cat#A11034 |
| Mouse anti-rabbit IgG HRP secondary | 1:1000 | Jackson Labs cat#211-035-109 |
| Goat anti-mouse IgG HRP secondary | 1:1000 | Jackson Labs cat#115-035-003 |
| DAPI Solution | 1:5000 | VWR cat#PK-CA70740043 |
