# Supplementary material for: Stem cell-derived brain-like endothelial cells to interrogate Streptococcus pneumoniae interaction with brain endothelium
Source: Virulence. 2025 Oct 3;16(1):2564281. doi: 10.1080/21505594.2025.2564281 (PMC12498544; doi:10.1080/21505594.2025.2564281)
Supplement: Supplemental Material [file KVIR_A_2564281_SM3956.zip › QVIR Supplement Table 2.docx]

Supplement Table 2: Primers used in this study

| **PCR** | **5’🡪3’** |
| --- | --- |
| CbpA up fwd | cgaatccttagcaatggcgg |
| CbpA up rev | atctcccattcttcccataacaagactg |
| CbpA down fwd | atcgataaggcaatgccaatggtgaatggg |
| CbpA down rev | cggagctggactcgttgtaa |
| Pilus up fwd | tgctttcctcttgtatgctga |
| Pilus up rev | atctcccattgtgtgtctccctgttaag |
| Pilus down fwd | atcgataagggcaggctgaagggaagac |
| Pilus down rev | ccaacgctcctcaagttctg |
| CbpA erm up fwd | ttatgggaagaatgggagataagacggttc |
| CbpA erm down rev | attggcattgccttatcgatacaaattccc |
| Pilus erm up fwd | ggagacacacaatgggagataagacggttc |
| Pilus erm down rev | ttcagcctgcccttatcgatacaaattccc |
| Ply up fwd | gcccttgctctggttaaaaa |
| Ply down rev | tagccgttttcttggctacg |
| SpxB up fwd | gaacagtcatgctgacaggag |
| SpxB down rev | acaaggattggagaaggaggtg |
| CapKO-1 | ggatctgcttgggaatacga |
| CapKO-2 | gtttgcttctaagtcttatttcccctttttcaagagcctcacg |
| CapKO-3 | gagtcgcttttgtaaatttggtatggggatgaatggaagga |
| CapKO-4 | tttcctgacaatgcaaatgg |
| **qPCR** |  |
| TJP1 fwd | gtccagaatctcggaaaagtgcc |
| TJP1 rev | ctttcagcgcaccataccaacc |
| Ocln fwd | atggcaaagtgaatgacaagcgg |
| Ocln rev | ctgtaacgaggctgcctgaagt |
| Cldn5 fwd | ctctgctggttcgccaacat |
| Cldn5 rev | cagctcgtacttctgcgaca |
| Snai1 fwd | tgccctcaagatgcacatccga |
| Snai1 rev | gggacaggagaagggcttctc |
| Vegfa fwd | ttgccttgctgctctacctcca |
| Vegfa rev | gatggcagtagctgcgctgata |
| IL6 fwd | agacagccactcacctcttcag |
| IL6 rev | ttctgccagtgcctctttgctg |
| IL8 fwd | gagagtgattgagagtggaccac |
| IL8 rev | cacaaccctctgcacccagttt |
| CXCL1 fwd | agcttgcctcaatcctgcatcc |
| CXCL1 rev | tccttcaggaacagccaccagt |
| CXCL2 fwd | ggcagaaagcttgtctcaaccc |
| CXCL2 rev | ctccttcaggaacagccaccaa |
| CCL20 fwd | aagttgtctgtgtgcgcaaatcc |
| CCL20 rev | ccattccagaaaagccacagtttt |
| GAPDH fwd | gtctcctctgacttcaacagcg |
| GAPDH rev | accaccctgttgctgtagccaa |
